# Supplementary material for: A pilot randomized controlled trial to explore the feasibility of a peer-delivered single-session brief intervention for youth with moderate risk substance use
Source: PLoS One. 2026 Mar 16;21(3):e0344661. doi: 10.1371/journal.pone.0344661 (PMC12991270; doi:10.1371/journal.pone.0344661)
Supplement: S1 File — (DOCX) [file pone.0344661.s001.docx]

Alcohol, Smoking & Substance Use Involvement Screening Test for Youth questionnaire (ASSIST-Y) scoring

|  | **Substance** | **Risk level** |
| --- | --- | --- |
|  | Tobacco products | 0 Not used  2-11 Moderate  12+ High |
|  | Alcoholic beverage | 0 Not used  2-17 Moderate  18+ High |
|  | Cannabis | 0 Not used  2-11 Moderate  12+ High |
|  | Cocaine | 0 Not used  2-8 Moderate  9+ High |
|  | Khat | 0 Not used  2-8 Moderate  9+ High |
|  | Inhalants | 0 Not used  2-8 Moderate  9+ High |
|  | Sedatives or Sleeping pills | 0 Not used  2-6 Moderate  7+ High |
|  | Hallucinogens | 0 Not used  2-8 Moderate  9+ High |
|  | Opioids | 0 Not used  2-6 Moderate  7+ High |
|  | Others- specify | 0 Not used  2-6 Moderate  7+ High |
