# Supplementary material for: A pilot randomized controlled trial to explore the feasibility of a peer-delivered single-session brief intervention for youth with moderate risk substance use
Source: PLoS One. 2026 Mar 16;21(3):e0344661. doi: 10.1371/journal.pone.0344661 (PMC12991270; doi:10.1371/journal.pone.0344661)
Supplement: S2 File — (DOCX) [file pone.0344661.s002.docx]

**FIDELITY RATING SCALE: Rater name:………………………………..Audio name………………………………….. Time taken………………………………….**

|  | **Did the peer** | **Not at all i.e. the peer did not do the step at all**  **(0)** | **Partially i.e.**  **the peer made an attempt but did not complete it/did not do it well)**  **(1)** | **The peer completed the step and did it well**  **(2)** | **Make comments on gaps for each step**  **Indicate NA if question not applicable** |
| --- | --- | --- | --- | --- | --- |
|  | **Brief intervention** | | | | |
|  | Peer asks youth if they are interested in seeing their ASSIST-Y scores |  |  |  |  |
|  | The peer presents the scores and level of risk associated with the substance in question |  |  |  |  |
|  | The peer communicates the risks associated with the highest scoring substance or the most problematic substance according to the youth**.** |  |  |  |  |
|  | Peer gives clear advice on reducing/ stopping use |  |  |  |  |
|  | Peer elicits responsibility *(‘you are ultimately responsible for your decisions…)* |  |  |  |  |
|  | Peer uses open ended questions to ask the clients about what they think about the scores |  |  |  |  |
|  | Peer reflects youths’ responses appropriately |  |  |  |  |
|  | Peer uses open-ended questions to ask the clients about how concerned they are |  |  |  |  |
|  | Peer reflects youths’ responses appropriately |  |  |  |  |
|  | Peer explores pros and cons of substance use using open-ended questions |  |  |  |  |
|  | Peer summarizes both sides |  |  |  |  |
|  | Peer asks youth how concerned they are about the bad things using an open-ended question |  |  |  |  |
|  | Peer summarizes and reflects the youths’ statements following Q22 |  |  |  |  |
|  | Peer explores readiness to change appropriately and elicits change talk |  |  |  |  |
|  | Peer provides menu of options/strategies for cutting/stopping as below |  |  |  |  |
|  | Peer encourages self-efficacy |  |  |  |  |
|  | Peer thanks the youth & gives them the appropriate take home information |  |  |  |  |
|  |  |  |  |  | **Total scores out of 34**  **……………………………** |
