# Supplementary material for: A pilot randomized controlled trial to explore the feasibility of a peer-delivered single-session brief intervention for youth with moderate risk substance use
Source: PLoS One. 2026 Mar 16;21(3):e0344661. doi: 10.1371/journal.pone.0344661 (PMC12991270; doi:10.1371/journal.pone.0344661)
Supplement: S4 File — (PDF) [file pone.0344661.s004.pdf]

Data Dictionary Codebook

Tunaweza Study (PID: 854)

09/26/2025 10:24am

**Instruments**	
**Instrument**	**Form Name**
Socio Demographics	socio_demographics
Lifetime Substance Use	assisty_scores
ASSIST-Y Scores	lifetime_substance_use
Baseline (PHQ-9)	baseline_phq9
Baseline (GAD-7) SCALE	baseline_gad7_scale
Baseline WHO-QOL BREF	baseline_whoqol_bref
3-month (PHQ-9)	month_phq9
3-month (GAD-7) SCALE	month_gad7_scale
3-month WHO-QOL BREF	month_whoqol_bref
Target Substance	target_substance

	#	Variable / Field Name	Field Label<br>*Field Note*	Field Attributes (Field Type, Validation, Choices, Calculations, etc.)
**Instrument: Socio Demographics (socio_demographics)**				
	1	[study_id]	Patient Study Number	text
	2	[date_today]	Date of data collection	text (date_ymd)<br>Field Annotation: @HIDEBUTTON
	3	[start_time]	Date and start time of the interview	text (datetime_ymd)<br>Field Annotation: @HIDEBUTTON
	4	[end_time]	Date and end time of the interview	text (datetime_ymd)<br>Field Annotation: @HIDEBUTTON
	5	[time_taken]	The time taken in minutes to interview the patient	calc<br>Calculation:<br>round(datediff([start_time], [end_time],"m","ymd",true), 0)
	6	[date_of_birth]	Section Header: *Socio- Demographics*<br>Date of birth<br>*YYYY-MM-DD*	text (date_ymd)<br>Field Annotation: @HIDEBUTTON
	7	[current_age]	The current age of the patient in years<br>*A calculated field in RedCap using today's date and the birth date of the patient.*	calc<br>Calculation: rounddown(datediff([date_of_birth], [date_today], "y", "ymd"), 0)
	8	[phone_contact]	Phone Contact	text
	9	[gender]	Gender	radio<br>1 Male<br>2 Female<br>3 Other (Specify): {other_gender}
	10	[other_gender]	Another type of gender	text

		Show the field ONLY if:<br>[gender] = '3'		
	11	[education_level]	Level of education	radio<br>1 No Primary<br>2 Incomplete primary<br>3 Complete primary<br>4 Incomplete secondary<br>5 Complete secondary<br>6 Tertiary +
	12	[marital_status]	Marital Status	radio<br>1 Never married<br>2 Separated/divorced/widowed<br>3 Married/cohabiting
	13	[living_arrangement]	Living arrangement; Lives with	radio<br>1 Family/relative<br>2 Friend/non-relative<br>3 Alone
	14	[parental_status]	Parental status	radio<br>1 Both parents alive<br>2 One parent alive (Mother)<br>3 One parent alive (Father)<br>4 Both parents died
	15	[hiv_status]	HIV status	radio<br>1 Positive<br>2 Negative<br>3 Unknown
	16	[type_of_intervention]	Type of intervention	radio<br>1 Brief intervention<br>2 Psychoeducation
	17	[fidelity_scores]	Fidelity Scores	text (number)
	18	[intervention_time]	The time taken for the intervention (in minutes)	text (number)
	19	[socio_demographics_complete]	Section Header: *Form Status*<br>Complete?	dropdown<br>0 Incomplete<br>1 Unverified<br>2 Complete

Instrument: Lifetime Substance Use (assisty_scores)

	20	[substance_abuse_base]	Substance Substance Use Yes/No a. Tobacco products {tobacco_use} b. Alcoholic beverage {alcohol_use} c. Cannabis {cannabis_use} d. Cocaine {cocaine_use} e. Miraa / Mogoka {miraa_mogoka_use} f. Inhalants {inhalant_use} g. Sedatives or Sleeping pills {sedative_use} h. Hallucinogens {hallucinogen_use} I. Opioids {opioid_use} j. Others- specify: {other_substance_abuse_use} {other_use}	descriptive
	21	[tobacco_use]	Tobacco products	yesno<br>1 Yes<br>0 No
	22	[alcohol_use]	Alcoholic beverage	yesno<br>1 Yes<br>0 No
	23	[cannabis_use]	Cannabis	yesno<br>1 Yes<br>0 No
	24	[cocaine_use]	Cocaine	yesno<br>1 Yes<br>0 No
	25	[miraa_mogoka_use]	Miraa / Mogoka	yesno<br>1 Yes<br>0 No
	26	[inhalant_use]	Inhalants	yesno<br>1 Yes<br>0 No
	27	[sedative_use]	Sedatives or Sleeping pills	yesno<br>1 Yes<br>0 No
	28	[hallucinogen_use]	Hallucinogens	yesno<br>1 Yes<br>0 No
	29	[opioid_use]	Opioids	yesno<br>1 Yes<br>0 No
	30	[other_use]	Others- specify	yesno<br>1 Yes<br>0 No

	31	[other_substance_abuse_use]	Another substance abuse	text
	32	[assisty_scores_complete]	Section Header: *Form Status*<br>Complete?	dropdown<br>0 Incomplete<br>1 Unverified<br>2 Complete
**Instrument: ASSIST-Y Scores (lifetime_substance_use)**				
	33	[substance_abuse]	Substance Baseline ASSIST-Y Scores 3 Months ASSIST-Y Scores a. Tobacco products {tobacco_score} {tobacco_3mo} b. Alcoholic beverage {alcohol_score} {alcohol_3mo} c. Cannabis {cannabis_score} {cannabis_3mo} d. Cocaine {cocaine_score} {cocaine_3mo} e. Miraa / Mogoka {miraa_mogoka_score} {miraa_mogoka_3mo} f. Inhalants {inhalant_score} {inhalant_3mo} g. Sedatives or Sleeping pills {sedative_score} {sedative_3mo} h. Hallucinogens {hallucinogen_score} {hallucinogen_3mo} I. Opioids {opioid_score} {opioid_3mo} j. Others-specify: {other_substance_abuse} {other_score} {other_3mo} TOTAL {total_assist_y_score} {total_assist_y_score_3mo}	descriptive
	34	[tobacco_score]	Tobacco products	text (number)
	35	[tobacco_3mo]	Tobacco products	text (number)
	36	[alcohol_score]	Alcoholic beverage	text (number)
	37	[alcohol_3mo]	Alcoholic beverage	text (number)
	38	[cannabis_score]	Cannabis	text (number)
	39	[cannabis_3mo]	Cannabis	text (number)
	40	[cocaine_score]	Cocaine	text (number)
	41	[cocaine_3mo]	Cocaine	text (number)
	42	[miraa_mogoka_score]	Miraa / Mogoka	text (number)
	43	[miraa_mogoka_3mo]	Miraa / Mogoka	text (number)
	44	[inhalant_score]	Inhalants	text (number)
	45	[inhalant_3mo]	Inhalants	text (number)
	46	[sedative_score]	Sedatives or Sleeping pills	text (number)
	47	[sedative_3mo]	Sedatives or Sleeping pills	text (number)
	48	[hallucinogen_score]	Hallucinogens	text (number)
	49	[hallucinogen_3mo]	Hallucinogens	text (number)

	50	[opioid_score]	Opioids	text (number)
	51	[opioid_3mo]	Opioids	text (number)
	52	[other_score]	Others- specify	text (number)
	53	[other_3mo]	Others- specify	text (number)
	54	[other_substance_abuse]	Another substance abuse	text
	55	[total_assist_y_score]	Baseline: Total ASSIST Y score	calc<br>Calculation: [tobacco_score]+ [alcohol_score]+[cannabis_score]+ [cocaine_score]+ [miraa_mogoka_score]+ [inhalant_score]+[sedative_score]+ [hallucinogen_score]+ [opioid_score]+ [other_score]
	56	[total_assist_y_score_3mo]	3 months: Total ASSIST Y score	calc<br>Calculation: [tobacco_3mo]+ [alcohol_3mo]+[cannabis_3mo]+ [cocaine_3mo]+ [miraa_mogoka_3mo]+ [inhalant_3mo]+[sedative_3mo]+ [hallucinogen_3mo]+ [opioid_3mo]+ [other_3mo]
	57	[lifetime_substance_use_complete]	Section Header: *Form Status*<br>Complete?	dropdown<br>0 Incomplete<br>1 Unverified<br>2 Complete
**Instrument: Baseline (PHQ-9) (baseline_phq9)**				
	58	[little_interest]	Section Header: *PATIENT HEALTH QUESTIONNAIRE (PHQ-9) Over the last 2 weeks, on how many days have you been bothered by any of the following problems? (Use "√ "to indicate your answer)*<br>Little interest or pleasure in doing things	radio (Matrix)<br>0 Not at all (0-1 day)<br>1 Several days (2-7 days over the past 2 weeks)<br>2 More than half of the days (8-12 days over the past 2 weeks)<br>3 Nearly every day (13-14 days over the past 2 weeks)
	59	[feeling_down]	Feeling down, depressed, or hopeless	radio (Matrix)<br>0 Not at all (0-1 day)<br>1 Several days (2-7 days over the past 2 weeks)<br>2 More than half of the days (8-12 days over the past 2 weeks)<br>3 Nearly every day (13-14 days over the past 2 weeks)
	60	[troubled_sleep]	Trouble falling or staying asleep, or sleeping too much	radio (Matrix)<br>0 Not at all (0-1 day)

				1 Several days (2-7 days over the past 2 weeks)<br>2 More than half of the days (8-12 days over the past 2 weeks)<br>3 Nearly every day (13-14 days over the past 2 weeks)
	61	[feeling_tired]	Feeling tired or having little energy	radio (Matrix)<br>0 Not at all (0-1 day)<br>1 Several days (2-7 days over the past 2 weeks)<br>2 More than half of the days (8-12 days over the past 2 weeks)<br>3 Nearly every day (13-14 days over the past 2 weeks)
	62	[poor_appetite]	Poor appetite or overeating	radio (Matrix)<br>0 Not at all (0-1 day)<br>1 Several days (2-7 days over the past 2 weeks)<br>2 More than half of the days (8-12 days over the past 2 weeks)<br>3 Nearly every day (13-14 days over the past 2 weeks)
	63	[feeling_bad]	Feeling bad about yourself- or that you are a failure or have let yourself or your family down	radio (Matrix)<br>0 Not at all (0-1 day)<br>1 Several days (2-7 days over the past 2 weeks)<br>2 More than half of the days (8-12 days over the past 2 weeks)<br>3 Nearly every day (13-14 days over the past 2 weeks)
	64	[trouble_concentrating]	Trouble concentrating on things, such as reading the newspaper or watching television	radio (Matrix)<br>0 Not at all (0-1 day)<br>1 Several days (2-7 days over the past 2 weeks)<br>2 More than half of the days (8-12 days over the past 2 weeks)<br>3 Nearly every day (13-14 days over the past 2 weeks)
	65	[moving_slowly]	Moving or speaking so slowly that other people could have noticed. Or the opposite - being so fidgety or restless that you have been moving around a lot more than usual	radio (Matrix)<br>0 Not at all (0-1 day)<br>1 Several days (2-7 days over the past 2 weeks)<br>2 More than half of the days (8-12 days over the past 2 weeks)

	#	Variable / Field Name	Field Label	Field Attributes
				3 Nearly every day (13-14 days over the past 2 weeks)
	66	[thoughts_better_dead]	Thoughts that you would be better off dead, or of hurting yourself	radio (Matrix)<br>0 Not at all (0-1 day)<br>1 Several days (2-7 days over the past 2 weeks)<br>2 More than half of the days (8-12 days over the past 2 weeks)<br>3 Nearly every day (13-14 days over the past 2 weeks)
	67	[total_score_phq_9]	Total Score: Patient Health Questionnaire (PHQ-9)	calc<br>Calculation: [little_interest]+[feeling_down]+[troubled_sleep]+[feeling_tired]+[poor_appetite]+[feeling_bad]+[trouble_concentrating]+[moving_slowly]+[thoughts_better_dead]
	68	[severity_level_depression]	Severity level: Patient Health Questionnaire (PHQ-9)	radio<br>1 1 - 4: Minimal depression<br>2 5 - 9: Mild depression<br>3 10 - 14: Moderate depression<br>4 15 - 19: Moderately severe depression<br>5 20 - 27:Severe depression
	69	[baseline_phq9_complete]	Section Header: *Form Status*<br>Complete?	dropdown<br>0 Incomplete<br>1 Unverified<br>2 Complete
**Instrument: Baseline (GAD-7) SCALE (baseline_gad7_scale)**				
	70	[feeling_nervous]	Section Header: *GENERALIZED ANXIETY DISORDER 7-ITEM (GAD-7) SCALE Over the last 2 weeks, how many days have you been bothered by the following problems?*<br>Feeling nervous, anxious,	radio (Matrix)<br>0 Not at all (0-1 day over the past 2 wks<br>1 Several days (2-7 days over the past 2 weeks)<br>2 Over half the days (8-12 days over the past 2 weeks)<br>3 Nearly every day (>12 days over the past 2 weeks)
	71	[worrying_non_stop]	Not being able to stop or control worrying	radio (Matrix)<br>0 Not at all (0-1 day over the past 2 wks<br>1 Several days (2-7 days over the past 2 weeks)

				2 Over half the days (8-12 days over the past 2 weeks)<br>3 Nearly every day (>12 days over the past 2 weeks)
	72	[different_things_worry]	Worrying too much about different things	radio (Matrix)<br>0 Not at all (0-1 day over the past 2 wks<br>1 Several days (2-7 days over the past 2 weeks)<br>2 Over half the days (8-12 days over the past 2 weeks)<br>3 Nearly every day (>12 days over the past 2 weeks)
	73	[trouble_relaxing]	Trouble relaxing	radio (Matrix)<br>0 Not at all (0-1 day over the past 2 wks<br>1 Several days (2-7 days over the past 2 weeks)<br>2 Over half the days (8-12 days over the past 2 weeks)<br>3 Nearly every day (>12 days over the past 2 weeks)
	74	[being_restless]	Being so restless that it is hard to sit still	radio (Matrix)<br>0 Not at all (0-1 day over the past 2 wks<br>1 Several days (2-7 days over the past 2 weeks)<br>2 Over half the days (8-12 days over the past 2 weeks)<br>3 Nearly every day (>12 days over the past 2 weeks)
	75	[easily_annoyed]	Becoming easily annoyed or irritable	radio (Matrix)<br>0 Not at all (0-1 day over the past 2 wks<br>1 Several days (2-7 days over the past 2 weeks)<br>2 Over half the days (8-12 days over the past 2 weeks)<br>3 Nearly every day (>12 days over the past 2 weeks)
	76	[feeling_afraid]	Feeling afraid as if something awful might happen	radio (Matrix)<br>0 Not at all (0-1 day over the past 2 wks<br>1 Several days (2-7 days over the past 2 weeks)

				2 Over half the days (8-12 days over the past 2 weeks)<br>3 Nearly every day (>12 days over the past 2 weeks)
	77	[total_score_phq_9v]	Total Score: GENERALIZED ANXIETY DISORDER 7-ITEM (GAD-7) SCALE	calc<br>Calculation: [feeling_nervous]+[worrying_non_stop]+[different_things_worry]+[trouble_relaxing]+[being_restless]+[easily_annoyed]+[feeling_afraid]
	78	[severity_level_anxiety]	Severity level: GENERALIZED ANXIETY DISORDER 7-ITEM (GAD-7) SCALE	radio<br>1 Score 0-4: Minimal Anxiety.<br>2 Score 5-9: Mild Anxiety.<br>3 Score 10-14: Moderate Anxiety.<br>4 Score greater than 15: Severe Anxiety.
	79	[baseline_gad7_scale_complete]	Section Header: *Form Status*<br>Complete?	dropdown<br>0 Incomplete<br>1 Unverified<br>2 Complete
**Instrument: Baseline WHO-QOL BREF (baseline_whoqol_bref)**				
	80	[instuctions]	Please read the question, assess your feelings, for the last two weeks, and circle the number on the scale for each question that gives the best answer for you.	descriptive
	81	[who_qol_bref_1]	How would you rate your quality of life in the last two weeks?	radio<br>1 Very poor<br>2 Poor<br>3 Neither poor nor good<br>4 Good<br>5 Very good
	82	[who_qol_bref_2]	How satisfied are you with your health in the last two weeks?	radio<br>1 Very dissatisfied<br>2 Fairly dissatisfied<br>3 Neither satisfied nor dissatisfied<br>4 Satisfied<br>5 Very satisfied
	83	[who_qol_bref_3]	Section Header: *The following questions ask about how much you have experienced certain things in the last two weeks.*<br>To what extent do you feel that physical pain prevents you from doing what you need to do in the last two weeks?	radio (Matrix)<br>1 Not at all<br>2 A Small Amount<br>3 A Moderate amount

				4 A great deal<br>5 An Extreme amount
	84	[who_qol_bref_4]	How much do you need any medical treatment to function in your daily life in the last two weeks?	radio (Matrix)<br>1 Not at all<br>2 A Small Amount<br>3 A Moderate amount<br>4 A great deal<br>5 An Extreme amount
	85	[who_qol_bref_5]	How much do you enjoy life in the last two weeks?	radio (Matrix)<br>1 Not at all<br>2 A Small Amount<br>3 A Moderate amount<br>4 A great deal<br>5 An Extreme amount
	86	[who_qol_bref_6]	To what extent do you feel your life to be meaningful in the last two weeks?	radio (Matrix)<br>1 Not at all<br>2 A Small Amount<br>3 A Moderate amount<br>4 A great deal<br>5 An Extreme amount
	87	[who_qol_bref_7]	Section Header: *The following questions ask about how much you have experienced certain things in the last two weeks.*<br>How well are you able to concentrate in the last two weeks?	radio (Matrix)<br>1 Not at all<br>2 Slightly<br>3 Moderately<br>4 Very<br>5 Extremely
	88	[who_qol_bref_8]	How safe do you feel in your daily life in the last two weeks?	radio (Matrix)<br>1 Not at all<br>2 Slightly<br>3 Moderately<br>4 Very<br>5 Extremely
	89	[who_qol_bref_9]	How healthy is your physical environment in the last two weeks?	radio (Matrix)<br>1 Not at all<br>2 Slightly<br>3 Moderately<br>4 Very<br>5 Extremely

	90	[who_qol_bref_10]	Section Header: *The following questions ask about how much you have experienced certain things in the last two weeks.*<br>Do you have enough energy for everyday life in the last two weeks?	radio (Matrix)<br>1 Not at all<br>2 Slightly<br>3 Somewhat<br>4 To a great extent<br>5 Completely
	91	[who_qol_bref_11]	Are you able to accept your bodily appearance in the last two weeks?	radio (Matrix)<br>1 Not at all<br>2 Slightly<br>3 Somewhat<br>4 To a great extent<br>5 Completely
	92	[who_qol_bref_12]	Have you enough money to meet your needs in the last two weeks?	radio (Matrix)<br>1 Not at all<br>2 Slightly<br>3 Somewhat<br>4 To a great extent<br>5 Completely
	93	[who_qol_bref_13]	How available to you is the information you need in your daily life in the last two weeks?	radio (Matrix)<br>1 Not at all<br>2 Slightly<br>3 Somewhat<br>4 To a great extent<br>5 Completely
	94	[who_qol_bref_14]	To what extent do you have the opportunity for leisure activities in the last two weeks?	radio (Matrix)<br>1 Not at all<br>2 Slightly<br>3 Somewhat<br>4 To a great extent<br>5 Completely
	95	[who_qol_bref_15]	How well are you able to get around physically in the last two weeks?	radio<br>1 Not at all<br>2 Slightly<br>3 Moderately<br>4 Very<br>5 Extremely
	96	[who_qol_bref_16]	Section Header: *The following questions ask you to say how good or satisfied you have felt about various aspects of your life over the last two weeks.*	radio (Matrix)<br>1 Very Dissatisfied

		How satisfied are you with your sleep in the last two weeks?	2 Fairly Dissatisfied<br>3 Satisfied nor Dissatisfied<br>4 Satisfied<br>5 Very satisfied
97	[who_qol_bref_17]	How satisfied are you with your ability to perform your daily living activities in the last two weeks?	radio (Matrix)<br>1 Very Dissatisfied<br>2 Fairly Dissatisfied<br>3 Satisfied nor Dissatisfied<br>4 Satisfied<br>5 Very satisfied
98	[who_qol_bref_18]	How satisfied are you with your capacity for work in the last two weeks?	radio (Matrix)<br>1 Very Dissatisfied<br>2 Fairly Dissatisfied<br>3 Satisfied nor Dissatisfied<br>4 Satisfied<br>5 Very satisfied
99	[who_qol_bref_19]	How satisfied are you with yourself in the last two weeks?	radio (Matrix)<br>1 Very Dissatisfied<br>2 Fairly Dissatisfied<br>3 Satisfied nor Dissatisfied<br>4 Satisfied<br>5 Very satisfied
100	[who_qol_bref_20]	How satisfied are you with your personal relationships in the last two weeks?	radio (Matrix)<br>1 Very Dissatisfied<br>2 Fairly Dissatisfied<br>3 Satisfied nor Dissatisfied<br>4 Satisfied<br>5 Very satisfied
101	[who_qol_bref_21]	How satisfied are you with your sex life in the last two weeks?	radio (Matrix)<br>1 Very Dissatisfied<br>2 Fairly Dissatisfied<br>3 Satisfied nor Dissatisfied<br>4 Satisfied<br>5 Very satisfied
102	[who_qol_bref_22]	How satisfied are you with the support you get from your friends in the last two weeks?	radio (Matrix)<br>1 Very Dissatisfied<br>2 Fairly Dissatisfied<br>3 Satisfied nor Dissatisfied

				4 Satisfied<br>5 Very satisfied
	103	[who_qol_bref_23]	How satisfied are you with the conditions of your living place in the last two weeks?	radio (Matrix)<br>1 Very Dissatisfied<br>2 Fairly Dissatisfied<br>3 Satisfied nor Dissatisfied<br>4 Satisfied<br>5 Very satisfied
	104	[who_qol_bref_24]	How satisfied are you with your access to health services in the last two weeks?	radio (Matrix)<br>1 Very Dissatisfied<br>2 Fairly Dissatisfied<br>3 Satisfied nor Dissatisfied<br>4 Satisfied<br>5 Very satisfied
	105	[who_qol_bref_25]	How satisfied are you with your transport in the last two weeks?	radio (Matrix)<br>1 Very Dissatisfied<br>2 Fairly Dissatisfied<br>3 Satisfied nor Dissatisfied<br>4 Satisfied<br>5 Very satisfied
	106	[how_often]	The following question refers to how often you have felt or experienced certain things in the last two weeks.	descriptive
	107	[who_qol_bref_26]	How often do you have negative feelings such as low mood, despair, anxiety, or depression, in the last two weeks?	radio<br>1 Never<br>2 Infrequently<br>3 Sometimes<br>4 Frequently<br>5 Always
	108	[total_score_qol]	Total Score: WHO-QOL BREF	calc<br>Calculation: [who_qol_bref_1]+[who_qol_bref_2]+[who_qol_bref_3]+[who_qol_bref_4]+[who_qol_bref_5]+[who_qol_bref_6]+[who_qol_bref_7]+[who_qol_bref_8]+[who_qol_bref_9]+[who_qol_bref_10]+[who_qol_bref_11]+[who_qol_bref_12]+[who_qol_bref_13]+[who_qol_bref_14]+[who_qol_bref_15]+[who_qol_bref_16]+[who_qol_bref_17]+[who_qol_bref_18]+

				[who_qol_bref_19]+ [who_qol_bref_20]+ [who_qol_bref_21]+ [who_qol_bref_22]+ [who_qol_bref_23]+ [who_qol_bref_24]+ [who_qol_bref_25]+[who_qol_bref_26]
	109	[baseline_whoqol_bref_complete]	Section Header: *Form Status*<br>Complete?	dropdown<br>0 Incomplete<br>1 Unverified<br>2 Complete
**Instrument: 3-month (PHQ-9) (month_phq9)**				
	110	[little_interest_3mo]	Section Header: *PATIENT HEALTH QUESTIONNAIRE (PHQ-9) Over the last 2 weeks, on how many days have you been bothered by any of the following problems? (Use "√ "to indicate your answer)*<br>Little interest or pleasure in doing things	radio (Matrix)<br>0 Not at all (0-1 day)<br>1 Several days (2-7 days over the past 2 weeks)<br>2 More than half of the days (8-12 days over the past 2 weeks)<br>3 Nearly every day (13-14 days over the past 2 weeks)
	111	[feeling_down_3mo]	Feeling down, depressed, or hopeless	radio (Matrix)<br>0 Not at all (0-1 day)<br>1 Several days (2-7 days over the past 2 weeks)<br>2 More than half of the days (8-12 days over the past 2 weeks)<br>3 Nearly every day (13-14 days over the past 2 weeks)
	112	[troubled_sleep_3mo]	Trouble falling or staying asleep, or sleeping too much	radio (Matrix)<br>0 Not at all (0-1 day)<br>1 Several days (2-7 days over the past 2 weeks)<br>2 More than half of the days (8-12 days over the past 2 weeks)<br>3 Nearly every day (13-14 days over the past 2 weeks)
	113	[feeling_tired_3mo]	Feeling tired or having little energy	radio (Matrix)<br>0 Not at all (0-1 day)<br>1 Several days (2-7 days over the past 2 weeks)<br>2 More than half of the days (8-12 days over the past 2 weeks)<br>3 Nearly every day (13-14 days over the past 2 weeks)

	114	[poor_appetite_3mo]	Poor appetite or overeating	radio (Matrix)<br>0 Not at all (0-1 day)<br>1 Several days (2-7 days over the past 2 weeks)<br>2 More than half of the days (8-12 days over the past 2 weeks)<br>3 Nearly every day (13-14 days over the past 2 weeks)
	115	[feeling_bad_3mo]	Feeling bad about yourself- or that you are a failure or have let yourself or your family down	radio (Matrix)<br>0 Not at all (0-1 day)<br>1 Several days (2-7 days over the past 2 weeks)<br>2 More than half of the days (8-12 days over the past 2 weeks)<br>3 Nearly every day (13-14 days over the past 2 weeks)
	116	[trouble_concentrating_3mo]	Trouble concentrating on things, such as reading the newspaper or watching television	radio (Matrix)<br>0 Not at all (0-1 day)<br>1 Several days (2-7 days over the past 2 weeks)<br>2 More than half of the days (8-12 days over the past 2 weeks)<br>3 Nearly every day (13-14 days over the past 2 weeks)
	117	[moving_slowly_3mo]	Moving or speaking so slowly that other people could have noticed. Or the opposite - being so fidgety or restless that you have been moving around a lot more than usual	radio (Matrix)<br>0 Not at all (0-1 day)<br>1 Several days (2-7 days over the past 2 weeks)<br>2 More than half of the days (8-12 days over the past 2 weeks)<br>3 Nearly every day (13-14 days over the past 2 weeks)
	118	[thoughts_better_dead_3mo]	Thoughts that you would be better off dead, or of hurting yourself	radio (Matrix)<br>0 Not at all (0-1 day)<br>1 Several days (2-7 days over the past 2 weeks)<br>2 More than half of the days (8-12 days over the past 2 weeks)<br>3 Nearly every day (13-14 days over the past 2 weeks)
	119	[total_score_phq_9_3mo]	Total Score: Patient Health Questionnaire (PHQ-9)	calc<br>Calculation: [little_interest_3mo]+ [feeling_down_3mo]+ [troubled_sleep_3mo]+ [feeling_tired_3mo]+

				[poor_appetite_3mo]+<br>[feeling_bad_3mo]+<br>[trouble_concentrating_3mo]+<br>[moving_slowly_3mo]+<br>[thoughts_better_dead_3mo]
	120	[severity_level_depression_3mo]	Severity level: Patient Health Questionnaire (PHQ-9)	radio<br>1 | 1 - 4: Minimal depression<br>2 | 5 - 9: Mild depression<br>3 | 10 - 14: Moderate depression<br>4 | 15 - 19: Moderately severe depression<br>5 | 20 - 27:Severe depression
	121	[month_phq9_complete]	Section Header: *Form Status*<br>Complete?	dropdown<br>0 | Incomplete<br>1 | Unverified<br>2 | Complete
**Instrument: 3-month (GAD-7) SCALE (month_gad7_scale)**				
	122	[feeling_nervous_3mo]	Section Header: *GENERALIZED ANXIETY DISORDER 7-ITEM (GAD-7) SCALE Over the last 2 weeks, how many days have you been bothered by the following problems?*<br>Feeling nervous, anxious,	radio (Matrix)<br>0 | Not at all (0-1 day over the past 2 wks<br>1 | Several days (2-7 days over the past 2 weeks)<br>2 | Over half the days (8-12 days over the past 2 weeks)<br>3 | Nearly every day (>12 days over the past 2 weeks)
	123	[worrying_non_stop_3mo]	Not being able to stop or control worrying	radio (Matrix)<br>0 | Not at all (0-1 day over the past 2 wks<br>1 | Several days (2-7 days over the past 2 weeks)<br>2 | Over half the days (8-12 days over the past 2 weeks)<br>3 | Nearly every day (>12 days over the past 2 weeks)
	124	[different_things_worry_3mo]	Worrying too much about different things	radio (Matrix)<br>0 | Not at all (0-1 day over the past 2 wks<br>1 | Several days (2-7 days over the past 2 weeks)<br>2 | Over half the days (8-12 days over the past 2 weeks)<br>3 | Nearly every day (>12 days over the past 2 weeks)

	125	[trouble_relaxing_3mo]	Trouble relaxing	radio (Matrix)<br>0 Not at all (0-1 day over the past 2 wks<br>1 Several days (2-7 days over the past 2 weeks)<br>2 Over half the days (8-12 days over the past 2 weeks)<br>3 Nearly every day (>12 days over the past 2 weeks)
	126	[being_restless_3mo]	Being so restless that it is hard to sit still	radio (Matrix)<br>0 Not at all (0-1 day over the past 2 wks<br>1 Several days (2-7 days over the past 2 weeks)<br>2 Over half the days (8-12 days over the past 2 weeks)<br>3 Nearly every day (>12 days over the past 2 weeks)
	127	[easily_annoyed_3mo]	Becoming easily annoyed or irritable	radio (Matrix)<br>0 Not at all (0-1 day over the past 2 wks<br>1 Several days (2-7 days over the past 2 weeks)<br>2 Over half the days (8-12 days over the past 2 weeks)<br>3 Nearly every day (>12 days over the past 2 weeks)
	128	[feeling_afraid_3mo]	Feeling afraid as if something awful might happen	radio (Matrix)<br>0 Not at all (0-1 day over the past 2 wks<br>1 Several days (2-7 days over the past 2 weeks)<br>2 Over half the days (8-12 days over the past 2 weeks)<br>3 Nearly every day (>12 days over the past 2 weeks)
	129	[total_score_phq_9v_3mo]	Total Score: GENERALIZED ANXIETY DISORDER 7-ITEM (GAD-7) SCALE	calc<br>Calculation: [feeling_nervous_3mo]+ [worrying_non_stop_3mo]+ [different_things_worry_3mo]+ [trouble_relaxing_3mo]+ [being_restless_3mo]+ [easily_annoyed_3mo]+ [feeling_afraid_3mo]
	130	[severity_level_anxiety_3mo]	Severity level: GENERALIZED ANXIETY DISORDER 7-ITEM (GAD-7) SCALE	radio<br>1 Score 0-4: Minimal Anxiety.

				2 Score 5-9: Mild Anxiety.<br>3 Score 10-14: Moderate Anxiety.<br>4 Score greater than 15: Severe Anxiety.
	131	[month_gad7_scale_complete]	Section Header: *Form Status*<br>Complete?	dropdown<br>0 Incomplete<br>1 Unverified<br>2 Complete
**Instrument: 3-month WHO-QOL BREF (month_whoqol_bref)**				
	132	[instuctions_3mo]	Please read the question, assess your feelings, for the last two weeks, and circle the number on the scale for each question that gives the best answer for you.	descriptive
	133	[who_qol_bref_1_3mo]	How would you rate your quality of life in the last two weeks?	radio<br>1 Very poor<br>2 Poor<br>3 Neither poor nor good<br>4 Good<br>5 Very good
	134	[who_qol_bref_2_3mo]	How satisfied are you with your health in the last two weeks?	radio<br>1 Very dissatisfied<br>2 Fairly dissatisfied<br>3 Neither satisfied nor dissatisfied<br>4 Satisfied<br>5 Very satisfied
	135	[who_qol_bref_3_3mo]	Section Header: *The following questions ask about how much you have experienced certain things in the last two weeks.*<br>To what extent do you feel that physical pain prevents you from doing what you need to do in the last two weeks?	radio (Matrix)<br>1 Not at all<br>2 A Small Amount<br>3 A Moderate amount<br>4 A great deal<br>5 An Extreme amount
	136	[who_qol_bref_4_3mo]	How much do you need any medical treatment to function in your daily life in the last two weeks?	radio (Matrix)<br>1 Not at all<br>2 A Small Amount<br>3 A Moderate amount<br>4 A great deal<br>5 An Extreme amount
	137	[who_qol_bref_5_3mo]	How much do you enjoy life in the last two weeks?	radio (Matrix)<br>1 Not at all

				2 A Small Amount<br>3 A Moderate amount<br>4 A great deal<br>5 An Extreme amount
	138	[who_qol_bref_6_3mo]	To what extent do you feel your life to be meaningful in the last two weeks?	radio (Matrix)<br>1 Not at all<br>2 A Small Amount<br>3 A Moderate amount<br>4 A great deal<br>5 An Extreme amount
	139	[who_qol_bref_7_3mo]	Section Header: *The following questions ask about how much you have experienced certain things in the last two weeks.*<br>How well are you able to concentrate in the last two weeks?	radio (Matrix)<br>1 Not at all<br>2 Slightly<br>3 Moderately<br>4 Very<br>5 Extremely
	140	[who_qol_bref_8_3mo]	How safe do you feel in your daily life in the last two weeks?	radio (Matrix)<br>1 Not at all<br>2 Slightly<br>3 Moderately<br>4 Very<br>5 Extremely
	141	[who_qol_bref_9_3mo]	How healthy is your physical environment in the last two weeks?	radio (Matrix)<br>1 Not at all<br>2 Slightly<br>3 Moderately<br>4 Very<br>5 Extremely
	142	[who_qol_bref_10_3mo]	Section Header: *The following questions ask about how much you have experienced certain things in the last two weeks.*<br>Do you have enough energy for everyday life in the last two weeks?	radio (Matrix)<br>1 Not at all<br>2 Slightly<br>3 Somewhat<br>4 To a great extent<br>5 Completely
	143	[who_qol_bref_11_3mo]	Are you able to accept your bodily appearance in the last two weeks?	radio (Matrix)<br>1 Not at all<br>2 Slightly<br>3 Somewhat

				4 To a great extent<br>5 Completely
	144	[who_qol_bref_12_3mo]	Have you enough money to meet your needs in the last two weeks?	radio (Matrix)<br>1 Not at all<br>2 Slightly<br>3 Somewhat<br>4 To a great extent<br>5 Completely
	145	[who_qol_bref_13_3mo]	How available to you is the information you need in your daily life in the last two weeks?	radio (Matrix)<br>1 Not at all<br>2 Slightly<br>3 Somewhat<br>4 To a great extent<br>5 Completely
	146	[who_qol_bref_14_3mo]	To what extent do you have the opportunity for leisure activities in the last two weeks?	radio (Matrix)<br>1 Not at all<br>2 Slightly<br>3 Somewhat<br>4 To a great extent<br>5 Completely
	147	[who_qol_bref_15_3mo]	How well are you able to get around physically in the last two weeks?	radio<br>1 Not at all<br>2 Slightly<br>3 Moderately<br>4 Very<br>5 Extremely
	148	[who_qol_bref_16_3mo]	Section Header: *The following questions ask you to say how good or satisfied you have felt about various aspects of your life over the last two weeks.*<br>How satisfied are you with your sleep in the last two weeks?	radio (Matrix)<br>1 Very Dissatisfied<br>2 Fairly Dissatisfied<br>3 Satisfied nor Dissatisfied<br>4 Satisfied<br>5 Very satisfied
	149	[who_qol_bref_17_3mo]	How satisfied are you with your ability to perform your daily living activities in the last two weeks?	radio (Matrix)<br>1 Very Dissatisfied<br>2 Fairly Dissatisfied<br>3 Satisfied nor Dissatisfied<br>4 Satisfied<br>5 Very satisfied

	150	[who_qol_bref_18_3mo]	How satisfied are you with your capacity for work in the last two weeks?	radio (Matrix)<br>1 Very Dissatisfied<br>2 Fairly Dissatisfied<br>3 Satisfied nor Dissatisfied<br>4 Satisfied<br>5 Very satisfied
	151	[who_qol_bref_19_3mo]	How satisfied are you with yourself in the last two weeks?	radio (Matrix)<br>1 Very Dissatisfied<br>2 Fairly Dissatisfied<br>3 Satisfied nor Dissatisfied<br>4 Satisfied<br>5 Very satisfied
	152	[who_qol_bref_20_3mo]	How satisfied are you with your personal relationships in the last two weeks?	radio (Matrix)<br>1 Very Dissatisfied<br>2 Fairly Dissatisfied<br>3 Satisfied nor Dissatisfied<br>4 Satisfied<br>5 Very satisfied
	153	[who_qol_bref_21_3mo]	How satisfied are you with your sex life in the last two weeks?	radio (Matrix)<br>1 Very Dissatisfied<br>2 Fairly Dissatisfied<br>3 Satisfied nor Dissatisfied<br>4 Satisfied<br>5 Very satisfied
	154	[who_qol_bref_22_3mo]	How satisfied are you with the support you get from your friends in the last two weeks?	radio (Matrix)<br>1 Very Dissatisfied<br>2 Fairly Dissatisfied<br>3 Satisfied nor Dissatisfied<br>4 Satisfied<br>5 Very satisfied
	155	[who_qol_bref_23_3mo]	How satisfied are you with the conditions of your living place in the last two weeks?	radio (Matrix)<br>1 Very Dissatisfied<br>2 Fairly Dissatisfied<br>3 Satisfied nor Dissatisfied<br>4 Satisfied<br>5 Very satisfied
	156	[who_qol_bref_24_3mo]	How satisfied are you with your access to health services in the last two weeks?	radio (Matrix)<br>1 Very Dissatisfied

				2 Fairly Dissatisfied<br>3 Satisfied nor Dissatisfied<br>4 Satisfied<br>5 Very satisfied
	157	[who_qol_bref_25_3mo]	How satisfied are you with your transport in the last two weeks?	radio (Matrix)<br>1 Very Dissatisfied<br>2 Fairly Dissatisfied<br>3 Satisfied nor Dissatisfied<br>4 Satisfied<br>5 Very satisfied
	158	[how_often_3mo]	The following question refers to how often you have felt or experienced certain things in the last two weeks.	descriptive
	159	[who_qol_bref_26_3mo]	How often do you have negative feelings such as low mood, despair, anxiety, or depression, in the last two weeks?	radio<br>1 Never<br>2 Infrequently<br>3 Sometimes<br>4 Frequently<br>5 Always
	160	[total_score_qol_3mo]	Total Score: WHO-QOL BREF	calc<br>Calculation: [who_qol_bref_1_3mo]+ [who_qol_bref_2_3mo]+ [who_qol_bref_3_3mo]+ [who_qol_bref_4_3mo]+ [who_qol_bref_5_3mo]+ [who_qol_bref_6_3mo]+ [who_qol_bref_7_3mo]+ [who_qol_bref_8_3mo]+ [who_qol_bref_9_3mo]+ [who_qol_bref_10_3mo]+ [who_qol_bref_11_3mo]+ [who_qol_bref_12_3mo]+ [who_qol_bref_13_3mo]+ [who_qol_bref_14_3mo] + [who_qol_bref_15_3mo]+ [who_qol_bref_16_3mo]+ [who_qol_bref_17_3mo]+ [who_qol_bref_18_3mo]+ [who_qol_bref_19_3mo]+ [who_qol_bref_20_3mo]+ [who_qol_bref_21_3mo]+ [who_qol_bref_22_3mo]+ [who_qol_bref_23_3mo]+ [who_qol_bref_24_3mo]+ [who_qol_bref_25_3mo]+ [who_qol_bref_26_3mo]
	161	[month_whoqol_bref_complete]	Section Header: *Form Status*<br>Complete?	dropdown<br>0 Incomplete

				1 Unverified<br>2 Complete

Instrument: Target Substance (target_substance)

	162	[target_substance]	BASELINE SUBSTANCE USE 3-MONTH SUBSTANCE USE Highest scoring or most problematic substance at Baseline Baseline ASSIST-Y Score for highest scoring or most problematic substance Baseline total ASSIST-Y scores 3-month ASSIST-Y Score for the highest scoring or most problematic substance 3-month total ASSIST-Y scores<br>{base_h_scored_subs}<br>{base_assist_y_score}<br>{base_total_assist_sco}<br>{mo_3_assist_y_score}<br>{m3_total_assist_sco}	descriptive
	163	[base_h_scored_subs]	Name of highest scoring or most problematic substance at baseline	text
	164	[base_assist_y_score]	Baseline ASSIST-Y Score for highest scoring or most problematic substance	text (number)
	165	[base_total_assist_sco]	Baseline total ASSIST-Y scores	text (number)
	166	[mo_3_assist_y_score]	3-month ASSIST-Y Score for the highest scoring or most problematic substance	text (number)
	167	[m3_total_assist_sco]	3-month total ASSIST-Y scores	text (number)
	168	[target_substance_complete]	Section Header: *Form Status*<br>Complete?	dropdown<br>0 Incomplete<br>1 Unverified<br>2 Complete
