# Supplementary material for: A pilot randomized controlled trial to explore the feasibility of a peer-delivered single-session brief intervention for youth with moderate risk substance use
Source: PLoS One. 2026 Mar 16;21(3):e0344661. doi: 10.1371/journal.pone.0344661 (PMC12991270; doi:10.1371/journal.pone.0344661)
Supplement: S7 File — (DOCX) [file pone.0344661.s007.docx]

Inclusivity in global research

PLOS’ policy on inclusivity in global research aims to improve transparency in the reporting of research performed outside of researchers’ own country or community and ensures that PLOS publications reporting global research adhere to high standards for research ethics and authorship. Authors of relevant research articles may be asked to complete the questionnaire below, which outlines ethical, cultural, and scientific considerations specific to inclusivity in global research. This questionnaire may be requested when researchers have travelled to a different country to conduct research, if research uses samples collected in another country, research with Indigenous populations or their lands, or if research is on cultural artefacts. Researchers travelling to another country solely to use laboratory equipment will not normally be required to complete the questionnaire. However, the questionnaire can be requested at the journal’s discretion for any submission – if you have been requested to complete this questionnaire by the PLOS journal you submitted to, please do so.

Please complete the questionnaire below and include this as a Supporting Information file with your manuscript. Note that if your paper is accepted for publication, this checklist will be published with your article in the supporting information files. Please ensure that you reference the checklist in the main body of your manuscript. We suggest adding a subsection ‘Inclusivity in global research’ to your Methods section and adding the following sentence: “Additional information regarding the ethical, cultural, and scientific considerations specific to inclusivity in global research is included in the Supporting Information (SX Checklist)”

The questions have been designed to be applicable to a wide range of study types, and there are subsections for both human subjects research and non-human subjects research. If any of the questions are not relevant to your research please mark them as “N/A” as appropriate.

**Ethical considerations, permits and authorship**

*This section is applicable to all research types.*

Provide details as to who granted permissions and/or consent for the study to take place in the Methods section of your manuscript. This should include the names of **all** ethics boards, governmental organizations, community leaders or other bodies that provided approval for the study. If individuals provided approval refer to these people by their role or title but do not list their name(s).
If there were any deviations from the study protocol after approval was obtained please provide details of these changes in the Methods section of your manuscript.

**Reported on page number 20.**

“Ethical approval to conduct the study was sought from the MTRH/ Moi University Institutional Research Ethics committee (IREC) and the Indiana University Institutional Review Board. All experimental protocols were approved by IREC and the Indiana University Institutional Review Board. Approval Number IREC220/2022.

Prior to data collection, written informed assent was sought from the youth aged 15-17 years. In addition, written informed consent was obtained from parents of youth aged 15-17 years. Written informed consent was sought from youth aged 18-24 years.”

**Reported on page number 6-7 under section on** “**Adjustments made to the trial after commencement”:**

**“**Because of practical challenges we faced during project implementation, we made three key adjustments to the protocol after commencement. These are summarized below:

(i) The initial plan was to recruit all participants then randomize to intervention or control. This led to significant dropout rates. We then decided to randomize at the point of recruitment. The details of both approaches have been described below.

(ii) Secondly, because we faced challenges with recruitment during this pilot, we amended the protocol and conducted three focus group discussions with 15 youths to obtain their perceptions on potential recruitment strategies for a future full-scale trial.

(ii) During the three-month follow-up, some youth had moved out of the study area and were unreachable. We therefore amended the protocol to include conducting the 3 month assessments online via Zoom.”

Did this study involve local collaborators that are residents of the country where the research was conducted or members of the community studied? If you do not have any authors from said communities, please provide an explanation for this below.

The study involved local investigators who are residents of the country (Kenya) where the research was conducted. The local investigators have been listed as authors in this manuscript: F.J. (first and corresponding author ), E.K., E.A., Y.O., J.B., G.K., and A.K.

Everyone listed as an author should meet PLOS’ criteria for authorship and all individuals who meet these criteria should be included in the author byline, rather than the acknowledgements. For further information please see the journal’s Authorship Policy.

Everyone listed as an author has met PLOS’ criteria for authorship.

**Human subjects research (e.g. health research, medical research, cross-cultural psychology)**

Did you obtain written informed consent from a representative of the local community or region before the research took place? How did you establish who speaks for the community? Details of written informed consent obtained from study participants should be reported separately in the Methods section of your manuscript.

Written informed consent was obtained from study participants. For minors, we obtained assent from the study participants and consent from the parents/guardians. This information has been **reported in the Ethics section page 20:**

“Prior to data collection, written informed assent was sought from the youth aged 15-17 years. In addition, written informed consent was obtained from parents of youth aged 15-17 years. Written informed consent was sought from youth aged 18-24 years.”

How did members of the local community provide input on the aims of the research investigation, its methodology, and its anticipated outcome(s)?

A section of the authors, who are members of the local community took part in research design. The local community took part in intervention adaptation.

When engaging with the local community, how did you ensure that the informed consent documents and other materials could be understood by local stakeholders?

The study tools and informed consent documents were translated to Swahili, the local language, to ensure that they were understandable by the study participants. The translation process has been reported on page 17:

“All study materials including the quantitative tools, FGD interview guides, the SSBI manual, and

the consent and assent forms were translated to Swahili. The need to translate the SSBI into Swahili was identified as a recommendation from the youth in a prior pilot study (32). The tools were first translated to Swahili by experienced translators with a good command of both the English and Swahili languages. These were then back translated to English by a separate set of translators. A team comprising translators, back-translators, and mental health experts discussed the translated documents and resolved any differences to arrive at the final Swahili versions. The translation process was guided by the WHO-Disability Assessment Schedule (WHO-DAS) 2.0 translation protocol (46).

Will the findings of the research be made available in an understandable format to stakeholders in the community where the study was conducted (e.g. via a presentation, summary report, copies of publications, etc.)? Please provide details of how this will be achieved.

The study fidnings will be presented to community members at a stakeholder feedback workshop using a simplified power-point presentation and pictures, as well as a summary report.

**Non-human subjects research using specimens/ animals collected as part of the study, or those housed in archival collections. Examples include archaeology, paleontology, botany and zoology.**

Did the permission you obtained from a local authority to perform the study include an agreement on access to outputs and benefit sharing? This may include procedures to enable fair distribution of the benefits and resources arising from the research performed. Please include any details of Prior Informed Consent and Benefit Sharing Agreements obtained. These may be required by field-specific regulations, for example the Convention on Biological Diversity (CBD) and the associated Nagoya Protocol.

Not applicable

If the material used in your study was imported, please A) provide the year it was imported and B) indicate whether permits were obtained to import/export the materials used, C) provide details of any permits obtained. If this information is not available, please indicate this.

Not applicable

If you used archival specimens, please state how the material used in your study was acquired by the institute it is held in and provide details of any permits obtained for the original excavations/ sample collection. If this information is not available, please indicate this.

Not applicable

How was the potential cultural significance of the materials collected in your study to local communities considered in your research design? Were Indigenous peoples and/or local researchers and institutions involved with archaeological excavations / collection of specimens? If so, please provide a description of their involvement.

Not applicable

If your manuscript includes photographs of human remains please indicate whether authors obtained permission from descendants or affiliated cultural communities to do so.

Not applicable
