# Supplementary material for: A pilot randomized controlled trial to explore the feasibility of a peer-delivered single-session brief intervention for youth with moderate risk substance use
Source: PLoS One. 2026 Mar 16;21(3):e0344661. doi: 10.1371/journal.pone.0344661 (PMC12991270; doi:10.1371/journal.pone.0344661)
Supplement: S8 File — (DOCX) [file pone.0344661.s008.docx]

**A pilot study to evaluate the feasibility & acceptability of a peer-provider delivered substance use screening and brief intervention for adolescents in Kenya**

**2. Project Summary/Abstract**

**Background:** Adolescent substance use is prevalent in Kenya and in the US, and is associated with significant negative health and social outcomes. Unfortunately adolescents in both regions have limited access to substance use treatment because services are costly and scarce. Substance use screening and brief Intervention (SBI) delivered in primary health care by peer-mentors, represents a promising strategy for overcoming these barriers to substance use treatment for adolescents.

**Objective:** The aim of this study is to pilot study procedures and obtain data on intervention acceptability, fidelity and preliminary efficacy, to determine the feasibility of a definitive randomized controlled trial (RCT) of the efficacy of a peer-delivered substance use SBI among adolescents attending an out-patient clinic in Kenya.

**Design:** The study will use both qualitative and quantitative methods to meet its objectives

**Intervention:** SBI includes a single session of screening using the Alcohol, Smoking & Substance Use Involvement Screening Test for Youth questionnaire (ASSIST-Y), followed by a brief intervention (20-30 minutes of motivational interviewing) for those with moderate and high risk substance use. We shall conduct five-day training for 3 peer-mentors on how to deliver the SBI. The peer-mentors will receive regular supervision throughout the study implementation process.

**Outcomes:** SBI feasibility will be defined by the extent to which fidelity to the SBI is maintained and the preliminary effects of the SBI on substance use and quality of life outcomes. SBI acceptability will be evaluated from the perspective of the adolescents using qualitative interviews guided by Sekhon’s theoretical framework of acceptability. Feasibility of conducting a future full-scale RCT will be explored by measuring outcomes such as study participation rate, willingness to be randomized, study completion rates and ability to measure effectiveness outcomes.

**Investigating team and future plans:** This study will be conducted by a team including faculty from Moi Teaching and Referral Hospital, faculty from Indiana University, and a team from the National Authority for Campaign Against Alcohol and drug abuse (NACADA). Our team has experience implementing and scaling peer-led substance use SBIs for adolescent populations. If the SBI and its delivery are found to be feasible and acceptable we plan to pilot it in Indiana as well as partner with the government to conduct a full-scale multi-site RCT.

**3. Technical Narrative** (No more than 6 pages)
**A. PROJECT DESCRIPTION** (Recommended 4 pages)
**1. Focus and significance of project.** Adolescence is a peak period for initiation of substance use (1). A global survey found the average age of onset for substance use to be 16-19 years (1). In the US, by the time high school students are seniors, almost 70% of them will have tried alcohol, half will have taken an illegal substance and close to 40% will have smoked a cigarette (2). In Kenya, a nationwide survey conducted by the National Authority for the Campaign Against Alcohol and drug Abuse (NACADA), found that 20% of adolescents had used at least one substance in their lifetime, and that the median age of onset was 11 years (3). Substance use among adolescents is associated with significant negative health and social consequences including poor educational outcomes (4), risky sexual behavior (5), negative mental health outcomes (6), neurocognitive deficits (7) and significant disability. In the 2010, Global Burden of Disease (GBD) study, substance use disorders (SUDs) were the second leading cause of disability among the mental and SUDs for children and adolescents aged 0-24 years (8). In that study, 7,173,000 DALYS were attributable to SUDs representing 25% of total DALYs from mental and SUDs in that age group (8). Even worse is that adolescent substance use is associated with faster progression to dependence (9). In the US, 15.2% of people who start drinking by age 14 eventually develop alcohol abuse or dependence (as compared to just 2.1% of those who initiate use after the age of 21 years) (10). Addressing adolescent substance use is therefore of high priority.

Unfortunately adolescents are often unable to access substance use treatment services (11,12). In Kenya, treatment for substance use is mainly offered by few privately-owned residential facilities (12). Services are therefore costly and scarce, and cannot be accessed by adolescents. In rural communities in Indiana in the US, adolescent substance use services are similarly scarce, and most of them do not deliver evidence-based interventions (13,14). Moreover, Indiana ranks 48^th^ worst in the US in terms of access to substance use services (13,14).

The World Health Organization (WHO) recommends screening and brief intervention (SBI) delivered in primary healthcare as a cost-effective population level intervention for identification and early intervention for risky substance use (15). In addition SBIs are considered best practice by the United Nations Office on Drugs and Crime (UNODC) in the prevention of substance use among adolescents (16). SBI is comprised of 2 parts: (i) **Screening**, which identifies substance use along a continuum, from no use to a high risk, using questions from a validated screening tool; (ii) **A brief intervention (BI),** is a short (15-30 minute) discussion between the healthcare provider (HCP) and the patient using motivational interviewing techniques. The goal of the BI is to encourage the patient to reduce/stop substance use in order to prevent health related consequences of harmful use (15). Primary HCP delivered SBI has been found to be effective in reducing low, moderate and high risk substance use among adolescents in the US (17,18), Czech Republic (17) and in South Africa (19).

While SBI was originally designed to be delivered by primary HCPs, the intervention could be delivered by lay providers especially in low-to-middle income countries (LMIC) where primary HCPs are few and face heavy workload (20). Peer-mentors are lay-providers (aged 18-24 years) found in adolescent clinics in Kenya, and present a potential means through which SBI may be delivered. Peer-mentors are well placed to deliver SBI because they already have basic training in counseling skills. Additionally, they are near age-mates with the adolescents, therefore can easily relate with them. Winn et al (21) found that it was feasible to deliver SBI for adolescents in the US using trained peer-mentors aged 18-28 years. To our knowledge, no study has evaluated the efficacy of a peer-mentor delivered SBI among adolescents in a LMIC. This pilot project seeks to explore the feasibility and acceptability of a peer-mentor delivered SBI for adolescents in Kenya, to inform the development of a large-scale future RCT. This project is in line with the Kenyan Ministry of Health (MOH) guidelines for delivery of adolescent friendly services (22) which lists substance use counseling as an essential service, and target 3.5 of the Sustainable Development Goals (SDGs) which requires that governments strengthen treatment and prevention of substance abuse.

**On-going work (Fogarty project):** Currently Dr. Jaguga is leading a research project whose aim is to evaluate the implementation outcomes of a peer-mentor delivered substance use SBI for adolescents (aged 15-24) attending an adolescent out-patient clinic at Moi Teaching & Referral Hospital in Eldoret, Kenya. The study will explore determinants of implementation from the perspective of peer-mentors, clinic staff, and adolescents using qualitative interviews guided by the Consolidated Framework for Implementation Research (CFIR), and using quantitative measures i.e. the dissemination and implementation science measures (23). Together, findings from the Fogarty project and results of the proposed work, will provide preliminary data in preparation for a full RCT funded by NIH, and ultimately to scale-up efforts to the US and other LMICs.

**2. Specific aims.** The overall goal of this project is to pilot study procedures and obtain data on SBI acceptability, fidelity and preliminary efficacy, in order to determine the feasibility of a definitive RCT of the efficacy of a peer-delivered substance use SBI among adolescents in Kenya. Specific aims include:

**Aim 1: Test the feasibility and acceptability of the SBI for adolescents with moderate and high risk substance use**

1. Using a single-blind pilot RCT, evaluate ability to capture outcome data and preliminarily assess the effect of the intervention on a) clinical care measures (substance use scores) and b) patient-centered outcomes (quality of life scores) for adolescents with moderate and high risk substance use.

2. Evaluate fidelity to the peer-mentor delivered SBI using checklists of key intervention components

3. Using qualitative methods, describe the acceptability of SBI from the perspective of the youth.

**Aim 2: Test the feasibility of the full-scale RCT by addressing questions such as can we recruit and successfully randomize our target population and are we able to retain study participants.**

**3. Innovation.**

**Use of peer mentors:** This project proposes to use peer-mentors to deliver a substance use SBI. Peer-mentors are youth aged 18-24 years with basic counseling skills who offer support to adolescents and are found in many adolescent out-patient settings. HCPs in LMICs and rural US often face heavy workload and are unable to deliver SBI. Peer-mentors represent a potentially sustainable and affordable strategy through which SBI may be delivered and this approach has not been piloted in an LMIC and in Indiana.

**Use of the Theoretical Framework for Acceptability to assess intervention acceptability:** This project proposes to use the Theoretical Framework for Acceptability (24) to assess for SBI acceptability from the perspective of youth. We chose this framework because it allows for comprehensive assessment of acceptability and assesses constructs not evaluated in the Fogarty project

**Substance use Screening and Brief Intervention:** The feasibility of implementing this intervention has not been well explored in LMICs, yet this intervention is cost-effective and efficacious, and has the potential to surmount many of the barriers to substance use treatment seen in LMICs.

**4. Approach (Project design and methods)**.

**Aim 1: Test the feasibility and acceptability of the SBI for adolescents with moderate and high risk substance use**

**1.2 Evaluate preliminary effects of the SBI for adolescents with moderate and high risk substance use**

**Study design:** Using a single-blind pilot RCT study design, we will evaluate the preliminary effectiveness of a peer-mentor delivered SBI in managing moderate and high risk substance use among adolescents aged 15-24 years. Participants will be randomly allocated in a 1:1 ratio to either the SBI intervention or the control condition. Reporting will be guided by the CONSORT checklist for pilot and feasibility trials (25).

**Setting:** We will recruit participants from the adolescent HIV clinic **(Rafiki clinic)** run by the Academic Model Providing Access to Health Care (AMPATH) (26). AMPATH is a large HIV care program in western Kenya and is a partnership between Moi Teaching and Referral Hospital (MTRH), North American Universities, and the Kenyan Ministry of Health. Rafiki clinic has a total enrolment of 800 adolescents (aged 15-24 years), offers mainly HIV care services, but also pre-exposure prophylaxis, contraceptive care, counseling, and recreational activities e.g. salsa dance. Eighty percent of adolescents attending the clinic are HIV positive. Three peer mentors aged 18-26 years, work full-time at Rafiki and have all received training on how to deliver SBI (as part of the Fogarty project).

**Participants:** Participants will be **adolescents attending the Rafiki clinic, aged 15-24 years** and who have moderate or high risk substance use as defined by the Alcohol, Smoking & Substance Use Involvement Screening Test for Youth questionnaire (ASSIST-Y) scores (28). We will exclude: (i) those ill during the appointment (ii) those who decline to assent/consent (ii) those already enrolled in the Fogarty project.

**Peer-mentors:** Three peer-mentors working full-time at Rafiki, and already trained in SBI will be eligible for participation in this study. The peer mentors, aged 21-26 years, received a 5-day training on SBI as part of the Fogarty project. For the proposed project, we will invite the peer-mentors to a 5-day refresher training which will be conducted by Dr. Jaguga and Aalsma, both of whom have extensive training and experience in conducting substance use interventions with adolescents. We will hold weekly supervision meetings with peer mentors to provide continual training on skills as needed and ensure fidelity to the intervention.

**Sample size:** For practical and budgetary considerations, because this is a pilot RCT, we will aim to have 25 participants per group (Total N=50). This sample size is large enough to inform us about the practicalities of delivering the control and SBI interventions. The proposed sample size is also in line with existing pilot RCT recommendations for proportionality to planned definitive RCT (i.e., we have ≥90% power to detect a small standardized effect size of 0.2 based on a 2-sided 5% level of significance) (27). We will recruit 36 participants per arm because we anticipate 20% attrition between recruitment and baseline data collection, and a further 20% attrition between baseline data collection and the 3-month data collection. The attrition rates are based on our prior pilot study experience. This sample size (N=60) also has been shown as a median sample size used for feasibility studies. While the primary purpose is not to make statistical inferences based on p-values, we conducted a power analysis given the sample size we plan to obtain in order to facilitate our interpretation of findings from our exploratory analyses. A sample size of 72 youth with moderate or high risk substance use can be feasibly recruited from Rafiki clinic over an 8-month period (assuming a 20% prevalence rate of moderate or high risk substance use [from our Fogarty work], an average clinic attendance rate of 60 per month and a study follow-up period of 3 months).

**Recruitment:** A trained research assistant (RA) will approach all adolescents presenting for care, confirm eligibility, explain study procedures and seek assent/consent in both English & Swahili. For youth aged 15-17 years, parental/guardian consent will be obtained in addition to youth assent. Consenting/assenting will be done in a private room within the clinic. The RA will administer the socio-demographic questionnaire as well as measures of depression and anxiety.

**Randomization*:*** Following enrollment and baseline assessment, participants will be randomized to one of the two study groups (allocation ratio: 1:1) - SBI intervention or the control condition. Stratified permuted block randomization procedures will be used to ensure between-group balance by sex and age. Randomization lists will be prepared by the study biostatistician and uploaded into a REDCap database to allow for concealed allocation among the study team while maintaining a blinded statistical team.

**Intervention:** Participants randomized to the intervention arm will undergo a **SBI** delivered by the peer-mentors. **Screening** will be performed using the ASSIST-Y (28). The BI will be delivered in a single session (20-30 minutes) using the FRAMES model i.e.(i) providing **feedback** on screening results (ii) ensuring **responsibility** on the part of the adolescents (iii) giving clear **advice** to stop/cut down (iv) giving **menu of options** (alternative healthy behaviors to engage in) (v) expressing **empathy,** and (vi) encouraging **self-**efficacy (15). The BI will be delivered for the highest scoring substance or the one the adolescent identifies as the most problematic. A manual to guide this intervention has been developed based on the WHO manual for ASSIST-linked BI as part of the Fogarty project.

**Control:** Participants assigned to the control arm will participate in a substance use education intervention. This intervention will entail review of material in the NACADA substance use education manual for adolescents and will be followed by a question-and-answer session. The manual contains summarized and simple information on the harms and myths related to alcohol, tobacco, cannabis, prescription medication and khat use, substances. This education intervention will be delivered over a single 20–30-minute session by a counselor stationed at Rafiki clinic.

**Preliminary effectiveness outcomes** will include change in substance use scores (measured using ASSIST-Y) and change in quality-of-life scores (measured using the Brief Version WHO-Quality of life tool) from baseline to 3 months post-intervention.

**1.2 Evaluate fidelity to the peer-mentor delivered SBI**

For the intervention arm of the study, fidelity to the SBI will be assessed by audio-recording of all sessions and rating them using a checklist of key elements of the SBI. Recordings will be independently rated by 2 members of the research team. Inter-rater reliability will be assessed between coders by calculating percent agreement.

**1.3 Using qualitative methods, describe the acceptability of SBI from the perspective of the youth.**

We will conduct semi-structured interviews with 25 purposively sampled youth to obtain information on SBI acceptability at the end of the 3-month SBI follow-up period. Interviews will draw on Sekhon’s theoretical framework of acceptability which consists of seven component constructs: affective attitude, burden, perceived effectiveness, ethicality, intervention coherence, opportunity costs, and self-efficacy (24).

**Aim 2: Test the feasibility of the full-scale RCT**

In order to test the feasibility of conducting a full-scale RCT, we will examine the following outcomes: study participation rate, proportion of participants meeting inclusion criteria who get excluded, proportion of participants willing to be randomized, study completion rate, participant burden, and data completeness.

Table 1 below outlines the measures we will be assessing to determine if our study trial protocols are feasible, and the benchmarks for feasibility.

We will also conduct four focus group discussions (FGDs) with 24 youth to explore the feasibility of various recruitment strategies in anticipation of a full-scale RCT.

| **Table 1. Feasibility Measures for Study Protocols** | | |  |  |
| --- | --- | --- | --- | --- |
| ***Measure*** | ***Definition*** | ***Administration time point*** | **Benchmarks to establish feasibility for conducting a full-scale randomized trial*** | **findings** |
| Study Participation Rate | Number of participants who consent to take part in the study divided by the number of eligible patients. We will also document reasons for refusal to participate in the study. | Ongoing | 80% of those who eligibility meet criteria consent to participate |  |
| Proportion of participants meeting inclusion criteria who get excluded | Number of participants excluded divided by number meeting inclusion criteria. We will document reasons for exclusion | Ongoing | 80% of those meeting inclusion criteria are not excluded |  |
| Proportion of participants willing to be randomized | Number of participants consenting to participate divided by number willing to be randomized to either study arm. | Ongoing | 80% of those consenting are willing to be randomized to either study arm |  |
| Study Completion Rate | Number of participants who complete both the baseline and month 3 assessments/intervention divided by the number of participants enrolled in each study arm | Baseline, month 3 | 80% complete both baseline and month 3 assessments |  |
| Participant Burden | Time required to complete data collection at each assessment time point. | Baseline, month 3 | 80% of participants complete study assessments and the SBI in less than 90 minutes at baseline; and study assessments in less than 60 minutes at month 3 |  |
| Data Completeness | Percentage of questionnaires/study measures completed | Baseline, month 3 | 80% of those who enroll will complete at least 80% of study questionnaires/measures |  |

**Quantitative data collection: A researcher designed questionnaire** will be used to collect socio-demographic data (age, sex, parental status, living arrangement, level of education) at baseline. The **Patient Health Questionnaire-9 (PHQ-9)** (31) will be used to collect data on depression at baseline. The PHQ-9 is a valid and reliable tool for measuring severity of major depression (31) and has been validated among Kenyan adolescents(30). The **Generalized Anxiety Disorder-7 (GAD-7) scale** (32) will be used to collect data on GAD at baseline. GAD-7 is a valid and reliable tool for measuring severity of GAD (32). Osborn et al 2019 (30) examined the psychometric properties of the GAD-7 among Kenyan adolescents and reported that the reliability was adequate. **ASSIST –Y questionnaire** will be used to assess for the level of substance use at baseline and month 3. ASSIST is a valid and reliable tool that asks about frequency of substance use in the past 3 months (28). The adolescents’ version of the tool was developed by experts from Adelaide University in Australia. The ASSIST-Y enquires about lifetime use of 9 substances (alcohol, tobacco, cannabis, cocaine, inhalants, amphetamines, opioids, hallucinogens and sedatives). Endorsement of lifetime use is followed by an assessment of substance use in the past 3 months. The level of substance involvement is categorized as moderate or high risk and cut-off scores vary for each substance. Unlike the adult version, the ASSIST-Y has no ‘low risk’ category. The ASSIST has been used in Kenya among youth (33). The **Brief Version of the WHO-Quality of Life [WHO-QOL BREF]** tool (29) will be used to assess for participant quality of life at baseline and month 3 (29). The tool is comprised of 26 questions organized into 4 domains: social relationships, environment, physical health, and psychological. The WHO-QOL BREF has been validated for use among adolescent populations (34).

**Qualitative data collection:**

***Semi-structured individual interviews:*** Perceptions on intervention acceptability will be obtained via semi-structured individual interviews with 25 purposively sampled youth who underwent the intervention. Representation of different age groups (15-17 years vs 18-24 years) and sex (male vs female) will be ensured among the sampled youth. Each identified youth will be invited to the interview via a phone call by the RA. Informed written consent/assent to participate in the interviews will be was sought from the youth by the RA at the time of recruitment. Interviews will be conducted in a private space within Rafiki clinic and are estimated to take about 1-1.5 hours. The interview guide will be developed based on Sekhon’s theoretical framework of acceptability which consists of seven component constructs: affective attitude, burden, perceived effectiveness, ethicality, intervention coherence, opportunity costs, and self-efficacy (24). The IDIs will be conducted by a facilitator who is not part of the research team and is experienced in qualitative data collection.

The interviews will be audio-recorded and field notes taken The interviews will be conducted at the end of the 3-month follow-up period at the time of the repeat assessment. The youth will be reimbursed 500/- for the repeat visit. This will cater for the extra time they will spend at the clinic completing the repeat assessments and taking part in the interviews. For youth who are far from Eldoret, we propose to conduct the interviews via an online meeting platform. For those, we will share with them data worth 1 GB to facilitate the interview.

***Focus Group Discussions:*** We propose to conduct four focus group discussions with 24 purposively sampled youth (6 youth per FGD). The goal of the FGDs will be to obtain information on the feasibility of recruiting youth with moderate risk substance use for a full-scale RCT. FGDs will be organized as follows, girls aged 15-17, girls aged 18-24, boys aged 15-17, and boys aged 18-24 years. Each identified youth will be invited to the discussion via a phone call by the RA. Informed written consent/assent to participate in the interviews was sought from the youth by the RA at the time of recruitment. Interviews will be conducted in a private space within Rafiki clinic and are estimated to take about 1.5-2 hours. The FGD guide will seek to obtain information on feasibility of various recruitment sites and strategies. The FGDs will be conducted by a facilitator who is not part of the research team and is experienced in qualitative data collection.

The discussions will be audio-recorded, and field notes taken. The discussions will be conducted at the end of the 3-month follow-up period at the time of the repeat assessment. The youth will be reimbursed 500/- for the repeat visit. This will cater for the extra time they will spend at the clinic completing the repeat assessments and taking part in the FGDs.

**Translation/adaptation of study material and the SBI manual**

All study material including the quantitative tools, qualitative interview guides, the SBI manual, and the consent and assent forms will be translated to Swahili. During the Fogarty project, the youth recommended that future interventions be conducted in Swahili. The tools will first be translated to Swahili by experienced translators with a good command of both the English and Swahili languages. The translators will identify problematic phrases. These will be back-translated to English by a separate set of translators. A team comprising of the translators, back-translators, and mental health experts will discuss the translated documents and resolve any differences to arrive at the final Swahili versions (WHODAS 2.0 translation protocol).

**Data management and confidentiality:** Study data will be recorded onto standardized paper study forms at the time of collection. Data will be anonymised by assigning each participant an unidentifiable study ID number at the time of enrolment, which will be used to identify them for all study materials. Paper data forms will immediately be filed and stored in a locked cabinet and signed study consent forms will be filed and stored separately from data forms to maintain participant anonymity. Study data will subsequently be entered into a secure database by an RA. Audio recordings of study sessions will be transferred onto a secure computer and deleted from their original recording device at the time of transfer. Data files and documents will be destroyed 7 years after the project is closed.

**Quantitative data analysis:** Descriptive statistics will be used to summarize socio-demographic and mental health characteristics of the adolescents. Means and standard deviations will be used to summarize continuous parametric data, medians and ranges for continuous non-parametric data, and frequencies and percentages for categorical data. The proportion of patients meeting each of the feasibility endpoints (eligibility, recruitment and attrition rates), with accompanying 95% CIs, will be calculated. Levels of fidelity will be obtained by calculating mean scores across all items of the fidelity checklist. We will perform item-level analysis to identify specific items driving low fidelity. Paired t-test will be used to compare the mean scores before and after the intervention. To compare the changes in the mean scores between the two arms, the Difference in Difference (DID) will be used.

**Qualitative data analysis:** Interviews will be transcribed then entered into NVivo for analysis. Data will be coded based on themes drawn from the theoretical framework of acceptability. Coded data will be reduced and synthesized using matrices structured by the main themes of the analysis.

**Ethical approval:** Ethical approval to conduct the study will be sought from the MTRH/ Moi University Institutional Research Ethics committee (IREC) and from Indiana University IRB. For participants aged 15-17 years, written informed consent will be obtained from parents/guardians and written assent will be sought from the youth. Written informed consent will be sought from participants aged 18 years and above. Informed consent and assent procedures will be conducted in both English and Swahili.

**5. Dissemination**. At the end of the study, we will conduct a one-day feedback workshop to disseminate study findings to key stakeholders (both government and non-governmental actors) in the fields of adolescent health and substance use. We will prepare a manuscript for peer review in a high impact journal, and a policy brief, targeting policy makers at the Kenyan MOH and NACADA. We will present our results at the Kenya Psychiatric Association, Kenya Pediatric Association, and the American Academy of Child and Adolescent Psychiatry scientific conferences.


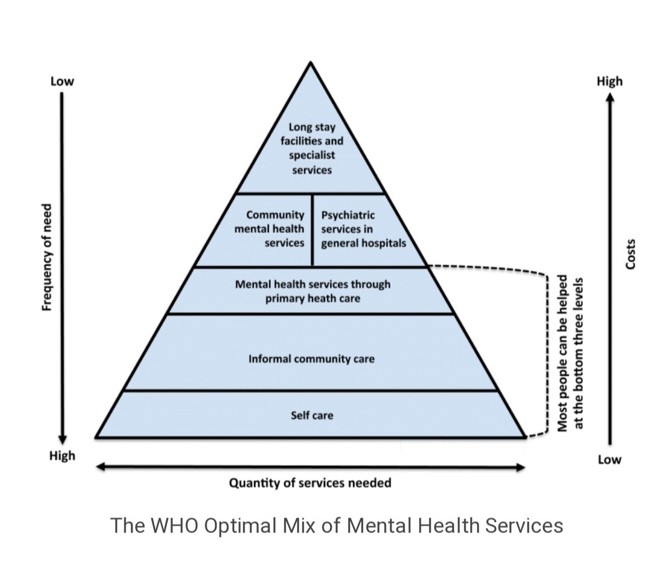


Fig 1: The WHO optimal mix of mental health services

**4. Proposed project timeline and milestones**The project will be conducted over a 24-month period. **Year 1:** The first 6 months **(Q1 and Q2)** will be spent seeking all the necessary ethical and administrative approvals, and conducting refresher training for the peer-mentors. Activities in **Q3 and 4 of year 1, and Q1 of year 2** will be spent in recruitment of adolescents and randomization to control/intervention arm; delivery of SBI or education intervention; baseline and month 3 data collection; piloting study procedures; weekly supervision meetings with peer-mentors; fidelity monitoring; conducting qualitative interviews on perceived acceptability of the SBI. Qualitative and quantitative data analysis will be conducted during **Q2 of year 2.** In Q3 and 4 of year 2, we will complete report writing and present study findings to key stakeholders, and embark on plans for a definitive RCT (Gantt chart below)

| **Table. Study Timeline / Milestones** | **Year 1** | | | | **Year 2** | | | |
| --- | --- | --- | --- | --- | --- | --- | --- | --- |
|  | **Q1** | **Q2** | **Q3** | **Q4** | **Q1** | **Q2** | **Q3** | **Q4** |
| **Start Up Activities:** (IRB and administrative approvals; clinicaltrials.gov registration, refresher training) | **X** | **X** |  |  |  |  |  |  |
| **Aim 1 & 2:** Recruitment of adolescents and randomization to control/intervention arm; delivery of SBI/education intervention; baseline and month 3 data collection; piloting study procedures; weekly supervision meetings with peer-mentors; fidelity monitoring; conducting qualitative interviews on perceived acceptability of the SBI |  |  | **X** | **X** | **X** |  |  |  |
| Qualitative & quantitative data analysis |  |  |  |  |  | **X** |  |  |
| Report writing, dissemination, full trial planning |  |  |  |  |  |  | **X** | **X** |

***COVID-19 considerations:*** Activities will take place outdoors or in well ventilated rooms with mask on for all participants. Hand washing and sanitizing facilities will be provided. Eating will not be permitted within the main study rooms. Seats will be arranged 1.5 meters apart to ensure social distancing. Our project will monitor changes in abide by all Kenyan laws and AMPATH research guidelines regarding social distancing.

**5. References/works cited**

1. Degenhardt L, Stockings E, Patton G, Hall WD, Lynskey M. The increasing global health priority of substance use in young people. The Lancet Psychiatry. 2016.

2. National Institute of Drug Abuse. Principles of Adolescent Substance Use Disorder Treatment: A Research-Based Guide: Introduction | NIDA [Internet]. [cited 2022 Feb 27]. Available from: https://nida.nih.gov/publications/principles-adolescent-substance-use-disorder-treatment-research-based-guide/introduction

3. National Aithority for the Campaign Against Alcohol and Drug Abuse. Status of Drugs and Substance Abuse among Primary School Pupils in Kenya. 2019. Available from:

4. Heradstveit O, Skogen JC, Hetland J, Hysing M. Alcohol and Illicit Drug Use Are Important Factors for School-Related Problems among Adolescents. Front Psychol [Internet]. 2017 Jun 20 [cited 2022 Jan 27];8(JUN):1023. Available from: http://journal.frontiersin.org/article/10.3389/fpsyg.2017.01023/full

5. Ritchwood TD, Ford H, DeCoster J, Lochman JE, Sutton M. Risky sexual behavior and substance use among adolescents: A meta-analysis. Child Youth Serv Rev [Internet]. 2015 May 1 [cited 2022 Jan 27];52:74–88. Available from: /pmc/articles/PMC4375751/

6. Gaitho D, Kumar M, Wamalwa D, Wambua GN, Nduati R. Understanding mental health difficulties and associated psychosocial outcomes in adolescents in the HIV clinic at Kenyatta National Hospital, Kenya. Ann Gen Psychiatry [Internet]. 2018 Jul 10 [cited 2020 Oct 8];17(1):29. Available from: /pmc/articles/PMC6038280/?report=abstract

7. Lisdahl KM, Gilbart ER, Wright NE, Shollenbarger S. Dare to delay? The impacts of adolescent alcohol and marijuana use onset on cognition, brain structure, and function. Front Psychiatry [Internet]. 2013 [cited 2022 Feb 2];4(JUL). Available from: /pmc/articles/PMC3696957/

8. Erskine HE, Moffitt TE, Copeland WE, Costello EJ, Ferrari AJ, Patton G, et al. A heavy burden on young minds: The global burden of mental and substance use disorders in children and youth. Psychol Med [Internet]. 2015 May 28 [cited 2022 Feb 3];45(7):1561–3. Available from: /pmc/articles/PMC5922255/

9. Richmond-Rakerd LS, Slutske WS, Wood PK. Age of initiation and substance use progression: A multivariate latent growth analysis. Psychol Addict Behav [Internet]. 2017 Sep 1 [cited 2020 Sep 2];31(6):664–75. Available from: /pmc/articles/PMC5593777/?report=abstract

10. samhsa. Results from the 2012 National Survey on Drug Use and Health : Summary of National Findings. 2012;

11. Zewdu S, Hanlon C, Fekadu A, Medhin G, Teferra S. Treatment gap, help-seeking, stigma and magnitude of alcohol use disorder in rural Ethiopia. Subst Abus Treat Prev Policy [Internet]. 2019 Jan 18 [cited 2020 Apr 28];14(1):4. Available from: https://substanceabusepolicy.biomedcentral.com/articles/10.1186/s13011-019-0192-7

12. Jaguga F, Kwobah E. A review of the public sector substance use disorder treatment and prevention systems in Kenya. Subst Abuse Treat Prev Policy. 2020;15(1).

13. Hoeft TJ, Fortney JC, Patel V, Unützer J. Task-Sharing Approaches to Improve Mental Health Care in Rural and Other Low-Resource Settings: A Systematic Review. J Rural Heal [Internet]. 2018 Dec 1 [cited 2022 Feb 21];34(1):48–62. Available from: /pmc/articles/PMC5509535/

14. Vesta. How Severe is the Shortage of Substance Abuse Specialists? | The Pew Charitable Trusts [Internet]. [cited 2022 Feb 21]. Available from: https://www.pewtrusts.org/en/research-and-analysis/blogs/stateline/2015/4/01/how-severe-is-the-shortage-of-substance-abuse-specialists

15. World Health Organisation. Brief Intervention. A Manual For Use In A Manual For Use In A Manual For Use In A Manual For Use In Primary Care Primary. 2003.

16. United Nations Office on drugs and Crime. International standards on drug use prevention - second updated edition [Internet]. 2018. 58 p. Available from: http://www.unodc.org/documents/prevention/standards_180412.pdf

17. Harris SK, Csémy L, Sherritt L, Starostova O, Van Hook S, Johnson J, et al. Computer-facilitated substance use screening and brief advice for teens in primary care: An international trial. Pediatrics [Internet]. 2012 Jun 1 [cited 2022 Feb 2];129(6):1072–82. Available from: /pediatrics/article/129/6/1072/32234/Computer-Facilitated-Substance-Use-Screening-and

18. Winters KC, Lee S, Botzet A, Fahnhorst T, Nicholson A. One-year outcomes and mediators of a brief intervention for drug abusing adolescents. Psychol Addict Behav [Internet]. 2014 [cited 2022 Jan 13];28(2):464–74. Available from: /pmc/articles/PMC4075470/

19. Carney T, Johnson K, Carrico A, Myers B. Acceptability and feasibility of a brief substance use intervention for adolescents in Cape Town, South Africa: A pilot study. Int J Psychol [Internet]. 2020 Dec 1 [cited 2022 Feb 2];55(6):1016–25. Available from: https://pubmed.ncbi.nlm.nih.gov/32285449/

20. Peltzer K, Matseke G, Azwihangwisi M, Babor T. Evaluation of alcohol screening and brief intervention in routine practice of primary care nurses in Vhembe district, South Africa. Croat Med J [Internet]. 2008 Jun [cited 2020 Sep 4];49(3):392–401. Available from: /pmc/articles/PMC2443624/?report=abstract

21. Winn LAP, Paquette KL, Donegan LRW, Wilkey CM, Ferreira KN. Enhancing adolescent SBIRT with a peer-delivered intervention: An implementation study. J Subst Abuse Treat. 2019 Aug 1;103:14–22.

22. Ministry of Health Kenya. Guidelines for Provision of Adolescent and Youth Friendly services in Kenya. 2016.

23. Hopkins J. Instrumentation - Dissemination & Scale Up - Global Mental Health - Centers and Institutes - Research - Johns Hopkins Bloomberg School of Public Health [Internet]. [cited 2020 Oct 10]. Available from: https://www.jhsph.edu/research/centers-and-institutes/global-mental-health/dissemination-and-scale-up/instrumentation/

24. Sekhon M, Cartwright M, Francis JJ. Acceptability of healthcare interventions: An overview of reviews and development of a theoretical framework. BMC Health Serv Res [Internet]. 2017 Jan 26 [cited 2022 Feb 21];17(1):88. Available from: http://bmchealthservres.biomedcentral.com/articles/10.1186/s12913-017-2031-8

25. Eldridge SM, Chan CL, Campbell MJ, Bond CM, Hopewell S, Thabane L, et al. CONSORT 2010 statement: extension to randomised pilot and feasibility trials. Pilot Feasibility Stud [Internet]. 2016 Dec 21 [cited 2022 Feb 20];2(1):64. Available from: http://pilotfeasibilitystudies.biomedcentral.com/articles/10.1186/s40814-016-0105-8

26. AMPATH [Internet]. Available from: https://www.ampathkenya.org/. Accessed 23. Feb 2022

27. Whitehead AL, Julious SA, Cooper CL, Campbell MJ. Estimating the sample size for a pilot randomised trial to minimise the overall trial sample size for the external pilot and main trial for a continuous outcome variable. Stat Methods Med Res [Internet]. 2016 Jun 1 [cited 2022 Feb 20];25(3):1057–73. Available from: /pmc/articles/PMC4876429/

28. Humeniuk R, Ali R, Babor TF, Farrell M, Formigoni ML, Jittiwutikarn J, et al. Validation of the alcohol, smoking and substance involvement screening test (ASSIST). Addiction [Internet]. 2008 Jun [cited 2020 Oct 25];103(6):1039–47. Available from: https://pubmed.ncbi.nlm.nih.gov/18373724/

29. Skevington SM, Lotfy M, O’Connell KA. The World Health Organization’s WHOQOL-BREF quality of life assessment: Psychometric properties and results of the international field trial a Report from the WHOQOL Group [Internet]. Vol. 13, Quality of Life Research. Qual Life Res; 2004 [cited 2022 Feb 20]. p. 299–310. Available from: https://pubmed.ncbi.nlm.nih.gov/15085902/

30. Osborn TL, Venturo-Conerly KE, Wasil AR, Schleider JL, Weisz JR. Depression and Anxiety Symptoms, Social Support, and Demographic Factors Among Kenyan High School Students. J Child Fam Stud [Internet]. 2020 May 1 [cited 2020 Oct 22];29(5):1432–43. Available from: https://link.springer.com/article/10.1007/s10826-019-01646-8

31. Kroenke K, Spitzer RL, Williams JB. The PHQ-9: validity of a brief depression severity measure. J Gen Intern Med [Internet]. 2001 Sep [cited 2018 Aug 17];16(9):606–13. Available from: http://www.ncbi.nlm.nih.gov/pubmed/11556941

32. Spitzer RL, Kroenke K, Williams JBW, Löwe B. A Brief Measure for Assessing Generalized Anxiety Disorder. Arch Intern Med [Internet]. 2006 May 22 [cited 2018 Aug 17];166(10):1092. Available from: http://www.ncbi.nlm.nih.gov/pubmed/16717171

33. Musyoka CM, Mbwayo A, Donovan D, Mathai M. Alcohol and substance use among first-year students at the University of Nairobi, Kenya: Prevalence and patterns. Francis JM, editor. PLoS One [Internet]. 2020 Aug 28 [cited 2020 Oct 22];15(8):e0238170. Available from: https://dx.plos.org/10.1371/journal.pone.0238170

34. Skevington SM, Dehner S, Gillison FB, McGrath EJ, Lovell CR. How appropriate is the WHOQOL-BREF for assessing the quality of life of adolescents? Psychol Heal [Internet]. 2014 Mar [cited 2022 Feb 20];29(3):297–317. Available from: https://pubmed.ncbi.nlm.nih.gov/24192254/

35. Gamarel KE, Brown L, Kahler CW, Fernandez MI, Bruce D, Nichols S. Prevalence and correlates of substance use among youth living with HIV in clinical settings. Drug Alcohol Depend [Internet]. 2016 Dec 1 [cited 2020 Sep 2];169:11–8. Available from: /pmc/articles/PMC5140709/?report=abstract

36. Atwoli L, Mungla PA, Ndung’u MN, Kinoti KC, Ogot EM. Prevalence of substance use among college students in Eldoret, western Kenya. BMC Psychiatry [Internet]. 2011 Feb 28 [cited 2020 Oct 19];11:34. Available from: /pmc/articles/PMC3053226/?report=abstract

37. Hogue A, Henderson CE, Ozechowski TJ, Robbins MS. Evidence Base on Outpatient Behavioral Treatments for Adolescent Substance Use: Updates and Recommendations 2007–2013. J Clin Child Adolesc Psychol [Internet]. 2014 Sep 1 [cited 2022 Jan 13];43(5):695–720. Available from: https://pubmed.ncbi.nlm.nih.gov/24926870/

38. Hogue A, Henderson CE, Becker SJ, Knight DK. Evidence Base on Outpatient Behavioral Treatments for Adolescent Substance Use, 2014–2017: Outcomes, Treatment Delivery, and Promising Horizons. J Clin Child Adolesc Psychol [Internet]. 2018 Jul 4 [cited 2022 Jan 13];47(4):499–526. Available from: /pmc/articles/PMC7192024/

39. World Health Organization (WHO). Optimal Mix of Health Services. 2007; Available from: https://www.who.int/mental_health/policy/services/2_Optimal Mix of Services_Infosheet.pdf

40. Aalsma MC, Dir AL, Zapolski TCB, Hulvershorn LA, Monahan PO, Saldana L, et al. Implementing risk stratification to the treatment of adolescent substance use among youth involved in the juvenile justice system: protocol of a hybrid type I trial. Addict Sci Clin Pract [Internet]. 2019 Sep 6 [cited 2022 Jan 13];14(1):36. Available from: https://ascpjournal.biomedcentral.com/articles/10.1186/s13722-019-0161-5

41. Aalsma MC, Aarons GA, Adams ZW, Alton MD, Boustani M, Dir AL, et al. Alliances to disseminate addiction prevention and treatment (ADAPT): A statewide learning health system to reduce substance use among justice-involved youth in rural communities. J Subst Abuse Treat. 2021 Sep 1;128:108368.

QUANTITATIVE TOOLS:

# SOCIO-DEMOGRAPHIC QUESTIONNAIRE for youth

**Patient Study Number:………………….. Date of recruitment:……………………………..**

**Date of birth…………….. Age………………………..Mobile no………………………………..**

**Gender**

Male Female Other

**Level of education**

No Primary

Incomplete primary

Complete primary

Incomplete secondary

Complete secondary

Tertiary +

**Marital status**

Never married Separated/divorced/widowed Married/cohabiting

**Living arrangement; Lives with**

Family/relative friend/non-relative Alone

**Parental status**

Both parents alive

One parent alive Specify which one Mother Father

Both parents died

# PHQ-9 QUESTIONNAIRE

| **Over the last 2 weeks, how often have you**  **been bothered by any of the following problems?**  *(Use “✔” to indicate your answer”* | **Never** | **1-3 days in a week** | | **4-5 days in a week** | **6-7 days in a week** |
| --- | --- | --- | --- | --- | --- |
| **1. Little interest or pleasure in doing things.......………** | **0** | **1** | | **2** | **3** |
| **2. Feeling down, depressed, or hopeless.………..……** | **0** | **1** | | **2** | **3** |
| 3. Trouble falling or staying asleep, or sleeping too much..................................................………..…….. | 0 | 1 | | 2 | 3 |
| 4. Feeling tired or having little energy......……...……… | 0 | 1 | | 2 | 3 |
| 5. Poor appetite or overeating.......................……….… | 0 | 1 | | 2 | 3 |
| 6. Feeling bad about yourself — or that you are a failure or have let yourself or your family down………………….. | 0 | 1 | | 2 | 3 |
| 7. Trouble concentrating on thing  s, such as reading the newspaper or watching television.……………………….. | 0 | 1 | | 2 | 3 |
| 8. Moving or speaking so slowly that other people could have noticed? Or the opposite — being so fidgety or restless that you have been moving .around a lot more than usual..............……………………………………………….. | 0 | 1 | | 2 | 3 |
| **9. Thoughts that you would be better off dead or of hurting yourself in some way......……………………………………** | **0** | | **1** | **2** | **3** |

# GAD-7 QUESTIONNAIRE
