# Supplementary material for: A pilot randomized controlled trial to explore the feasibility of a peer-delivered single-session brief intervention for youth with moderate risk substance use
Source: PLoS One. 2026 Mar 16;21(3):e0344661. doi: 10.1371/journal.pone.0344661 (PMC12991270; doi:10.1371/journal.pone.0344661)
Supplement: Attachment — Submitted filename: Resubmission response to reviewer comments.docx [file pone.0344661.s010.docx]

Dr. Florence Jaguga

Moi Teaching & Referral Hospital

P.O. BOX 3-30100

**ELDORET, KENYA**

19^th^ January 2026

Emily Chenette

Editor-in-Chief,

Plos One

Dear Madam,

**RESPONSE TO REVIEWERS’ COMMENTS**

We wish to submit a response to reviewers’ comments for the manuscript entitled **“A pilot randomized controlled trial (RCT) to explore the feasibility of a peer-delivered single-session brief intervention for youth with moderate risk substance use.”**

The authors thank the editor and reviewers for their comments.

A point-by-point response is provided below.

The page and line numbers refer to the marked-up copy of the revised manuscript (i.e., the version with tracked changes).

Sincerely,

**Florence Jaguga (MBchB, MMed Psychiatry)**

**Response to editor’s and reviewers’ comments**

**Editor’s comments:**

**1. Please ensure that your manuscript meets PLOS ONE's style requirements, including those for file naming.**

**Response:** We have aligned the manuscript including author details and file naming with PLOS ONE's style requirements.

**2. Please include a complete copy of PLOS’ questionnaire on inclusivity in global research in your revised manuscript.**

**Response:** A completed version of the questionnaire has been provided as Supporting Information- Supporting File 6.

**3. Thank you for stating the following financial disclosure: This project was funded, in part, with support from the Indiana Clinical and Translational Sciences Institute funded, in part by Grant Number UL1TR002529 from the National Institutes of Health, National Center for Advancing Translational Sciences, Clinical and Translational Sciences Award.
Please state what role the funders took in the study. If the funders had no role, please state: "The funders had no role in study design, data collection and analysis, decision to publish, or preparation of the manuscript." If this statement is not correct you must amend it as needed. Please include this amended Role of Funder statement in your cover letter; we will change the online submission form on your behalf.**

**Response:** The authors confirm that the funders had no role in study design, data collection and analysis, decision to publish, or preparation of the manuscript. The revised financial disclosure statement therefore reads as below. Please change it on the online submission on the authors’’ behalf:

“This project was funded, in part, with support from the Indiana Clinical and Translational Sciences Institute funded, in part by Grant Number UL1TR002529 from the National Institutes of Health, National Center for Advancing Translational Sciences, Clinical and Translational Sciences Award. The funders had no role in study design, data collection and analysis, decision to publish, or preparation of the manuscript." This amendment has been included in the resubmission cover letter.

**4. Thank you for stating in your Funding Statement: This project was funded, in part, with support from the Indiana Clinical and Translational Sciences Institute funded, in part by Grant Number UL1TR002529 from the National Institutes of Health, National Center for Advancing Translational Sciences, Clinical and Translational Sciences Award. Please provide an amended statement that declares *all* the funding or sources of support (whether external or internal to your organization) received during this study, as detailed online in our guide for authors at** [**http://journals.plos.org/plosone/s/submit-now**](http://journals.plos.org/plosone/s/submit-now)**.  Please also include the statement “There was no additional external funding received for this study.” in your updated Funding Statement. Please include your amended Funding Statement within your cover letter. We will change the online submission form on your behalf.**

**Response:** The authors have detailed all sources of funding and aligned the financial disclosure statement with journal requirements. Revised statement below. Please change it on the online submission on the authors’ behalf:

“This project was funded, in part, with support from the Indiana Clinical and Translational Sciences Institute (https://indianactsi.org/) funded, in part by Grant Number UL1TR002529 from the National Institutes of Health, National Center for Advancing Translational Sciences, Clinical and Translational Sciences Award. The Grant was awarded to M.A. The funders had no role in study design, data collection and analysis, decision to publish, or preparation of the manuscript. There was no additional external funding received for this study.”

**5. Your ethics statement should only appear in the Methods section of your manuscript. If your ethics statement is written in any section besides the Methods, please delete it from any other section.**

**Response:** The ethics statement now only appears in the Methods section of the manuscript page 20

**6. Please include captions for your Supporting Information files at the end of your manuscript, and update any in-text citations to match accordingly. Please see our Supporting Information guidelines for more information:** [**http://journals.plos.org/plosone/s/supporting-information**](http://journals.plos.org/plosone/s/supporting-information)**.**

**Response:** This has been done see page 37-38

**7. If the reviewer comments include a recommendation to cite specific previously published works, please review and evaluate these publications to determine whether they are relevant and should be cited.**

**Response:** There is no requirement by the reviewers to cite specific previously published works.

**8. Please review your reference list to ensure that it is complete and correct. If you have cited papers that have been retracted, please include the rationale for doing so in the manuscript text, or remove these references and replace them with relevant current references. Any changes to the reference list should be mentioned in the rebuttal letter that accompanies your revised manuscript. If you need to cite a retracted article, indicate the article’s retracted status in the References list and also include a citation and full reference for the retraction notice.**

**Response:** The authors confirm that the reference list is complete and correct

**Reviewer #1:**

A well conducted study. Comprehensive presentation has been made of both quantitative and qualitative data. There is clear alignment with feasibility trial objectives.
Standardized and validated measures (PHQ-9, GAD-7, WHO-QOL BREF, ASSIST-Y) have been used. There is methodological transparency in recruitment and fidelity monitoring.
This study makes a valuable contribution to adolescent substance use intervention research in low-resource settings and is methodologically rigorous and well-reported.

**Response:** The authors thank the reviewer for this comment.

**Reviewer #2:**

**1. Method wise, the study is okay. Explanations are given appropriately.**

**Response:** The authors thank the reviewer for this comment.

**2. Just a minor change, In Table 4, write Number of participants outside brackets and percentages in brackets e.g. 25 (78.1%) and NOT 78.1% (25). Make it uniform as shown in other tables.**

**Response:** This has been corrected see Table 6 page 21.

**Reviewer #3:**

**Here is a list of specific comments. Note: line and page numbering in reviews and comments is based on ruler applied in Editorial Manager-generated PDF.**

**1. Page 2, lines 54–56: I recommend reporting their effect sizes and their 95% CIs.**

**Response:** For the effect sizes, we have reported the standardized mean differences and corresponding confidence intervals in the abstract as below: (line 52-57 page 2).

“The SSBI showed a small effect on reducing total ASSIST-Y (Standardized Mean Difference [SMD] -0.33 95% Confidence Interval [CI] -0.83,0.16) scores in the intervention group compared to the control. There was a moderate improvement in the quality of life for the intervention group compared to the control (SMD -0.41 CI -0.91,0.09). The intervention had no effect on depression (SMD 0.23 CI -0.27,0.72) and anxiety symptoms (SMD 0.70 CI 0.19,1.2) at month 3”.

**2. Page 4, line 112: The term, brief interventions, is quite general. I recommend describing the brief interventions, especially the trial’s interventions, more specifically.**

**Response:** In the introduction section we have now provided a general description of brief interventions on page 3 line 76-78, and a description of the trial brief intervention on page 6 line 132-136.

**Description of brief interventions broadly (page 3 line 76-80)**

“Brief interventions (BIs) for substance use are recommended as a public health strategy for prevention and early intervention for addressing youth substance use problems (14,15). BIs typically comprise of screening to determine level of substance involvement, followed by counseling based on motivational interviewing principles (16). The intervention typically lasts 1-4 sessions (14,17).”

**A brief description of the trial brief intervention** which is a single-session brief intervention is provided in the introduction (page 5 line 127-130).

“The single-session brief intervention was adapted from the WHO Alcohol, Smoking & Substance Use Involvement Screening Test (ASSIST)-linked BI manual for use in primary care and is based on Feedback Responsibility Advice Menu of options Empathy Self-efficacy (FRAMES) model and motivational interviewing principles.”

A detailed description of the single session brief intervention is provided in the methods section page 11 lines 266-288 and Table 1 page 13.

In addition, we have refined the manuscript title to read “A pilot randomized controlled trial to explore the feasibility of a peer-delivered **single-session brief intervention** for youth with moderate risk substance use”.

**3. The brief intervention is not fully introduced until pages 13–14. I recommend briefly introducing them in the Introduction section.**

**Response:** A brief description of the trial brief intervention has been provided in the introduction section on page 5 line 127-130. See response no. 2 above.

**3. Page 8, lines 199–201: I recommend including the ASSIST-Y scores that are used to determine moderate and high risks. [After reading further, I understand that it is impossible to list the cutoffs for all 9 substances in the abstract. Please ignore the aforementioned comment and consider including a supplementary table listing the substance-specific cutoffs for moderate and high risks.]**

**Response:** ASSIST-Y scoring defining moderate and high risk is provided below. ASSIST-Y has no low-risk category. A file with ASSIST-Y scoring has been added as **Supporting file 7** and has been referenced in the manuscript page 17 lines 364-366.

|  | **Substance** | **Risk level** |
| --- | --- | --- |
| 1. | Tobacco products | 0 Not used  2-11 Moderate  12+ High |
| 2. | Alcoholic beverage | 0 Not used  2-17 Moderate  18+ High |
| 3. | Cannabis | 0 Not used  2-11 Moderate  12+ High |
| 4. | Cocaine | 0 Not used  2-8 Moderate  9+ High |
| 5. | Khat | 0 Not used  2-8 Moderate  9+ High |
| 6. | Inhalants | 0 Not used  2-8 Moderate  9+ High |
| 7. | Sedatives or Sleeping pills | 0 Not used  2-6 Moderate  7+ High |
| 8. | Hallucinogens | 0 Not used  2-8 Moderate  9+ High |
| 9. | Opioids | 0 Not used  2-6 Moderate  7+ High |
| 10. | Others- specify | 0 Not used  2-6 Moderate  7+ High |

**4. Page 8, lines 203–204: Is this a subjective measure or an evident-based measure? If the latter, I recommend including relevant references.**

**Response:** The sample size of 25 is based on existing recommendations for pilot trial sample sizes. Whitehead et al recommend that for a main trial designed with 90% power and two-sided 5% significance, and small effect size, the pilot trial sample sizes per treatment arm should be 25. Reference below.

Whitehead AL, Julious SA, Cooper CL, Campbell MJ. Estimating the sample size for a pilot randomised trial to minimise the overall trial sample size for the external pilot and main trial for a continuous outcome variable. Stat Methods Med Res [Internet]. 2016 Jun 1 [cited 2024 May 26];25(3):1057. Available from: /pmc/articles/PMC4876429/

The small effect size is based on evidence from studies evaluating effectiveness of BI among youth (references below).

Tanner-Smith EE, Lipsey MW. Brief alcohol interventions for adolescents and young adults: a systematic review and meta-analysis. J Subst Abuse Treat [Internet]. 2015 Apr 1 [cited 2024 Apr 28];51:1–18. Available from: https://pubmed.ncbi.nlm.nih.gov/25300577/

Carney T, Myers B. Effectiveness of early interventions for substance-using adolescents: Findings from a systematic review and meta-analysis [Internet]. Vol. 7, Substance Abuse: Treatment, Prevention, and Policy. BioMed Central; 2012 [cited 2020 Oct 20]. p. 25. Available from: https://substanceabusepolicy.biomedcentral.com/articles/10.1186/1747-597X-7-25

A paragraph describing this has been included in the revised manuscript page 7-8 line 172-187.

“We aimed to have 25 participants per group (Total N=50). This sample size is large enough to inform us about the practicalities of delivering the control and the SSBI, and is in line with existing pilot RCT recommendations for proportionality to planned definitive RCT(41) (i.e., we have ≥90% power to detect a small, standardized effect size of 0.2 [an effective SSBI will result in a 20% difference in the reduction in the total ASSIST-Y scores between SSBI and psychoeducation arms]) (41). The small, standardized effect size is consistent with meta-analytic evidence from studies evaluating the effectiveness of BIs among adolescents and youth, which demonstrates 10–20% reductions in substance use.(25,42).”

**5. Page 9, lines 206–207: I recommend describing which efficacy (secondary) outcomes will satisfy the standardized effect size of 0.2 in the target population.**

**Response:** An effective SSBI will result in a 20% difference in the reduction in the total ASSIST-Y scores between SSBI and psychoeducation arms. We have described this on Page 7 line 176-177.

“We aimed to have 25 participants per group (Total N=50). This sample size is large enough to inform us about the practicalities of delivering the control and the SSBI, and is in line with existing pilot RCT recommendations for proportionality to planned definitive RCT(41) (i.e., we have ≥90% power to detect a small, standardized effect size of 0.2 [an effective SSBI will result in a 20% difference in the reduction in the total ASSIST-Y scores between SSBI and psychoeducation arms]) (41).”

**6. Page 9, lines 227–228: I recommend specifying the block size and the age categories (I assume the stratification factor of age is not continuous).**

**Response:** Yes, the stratification factor of age is not continuous. We have provided the block sizes that were achieved by stratum on Table 2 (page 11).

**Table 2: Block sizes achieved by stratum**

|  | **Single-Session Brief Intervention** | | **Psychoeducation** | |
| --- | --- | --- | --- | --- |
|  | **Male** | **Female** | **Male** | **Female** |
| **15-17 years** | 7 | 3 | 5 | 2 |
| **18-24 years** | 17 | 11 | 16 | 9 |

**7. Page 16, line 401: I recommend replacing “parametric” with ‘normally distributed.’**

**Response:** Parametric has been replaced with normally distributed on page 19 line 413-414.

“Means and standard deviations were used to summarize continuous normally distributed data, medians and ranges for continuous non-normally distributed data, and frequencies and percentages for categorical data.”

**8. Page 18, Table 4: It is surprising to see many imbalance in baseline variables (Tables 4–7).**

**Response:** Baseline imbalances may arise by chance, particularly in studies with small sample sizes, despite appropriate randomization (reference below). We have included this as a limitation (page 34, line 720-723).

“... we had baseline imbalances between the intervention and control groups likely due to the small sample size (54). Accordingly, the SMDs from this study should be interpreted with caution, and a definitive trial with adequate sample size is needed to determine the effectiveness of the SSBI.”

Chu R, Walter SD, Guyatt G, Devereaux PJ, Walsh M, Thorlund K, Thabane L. Assessment and implication of prognostic imbalance in randomized controlled trials with a binary outcome--a simulation study. PLoS One. 2012;7(5):e36677. doi: 10.1371/journal.pone.0036677. Epub 2012 May 22. PMID: 22629322; PMCID: PMC3358303.

**9. Figure 1 (CONSORT Flow chart)
(9a) The text in the first side table is truncated.**

**Response:** This has been corrected

**(9b) I recommend adding another box before Randomized for Eligible even if the number of eligible is the same as the number of randomized.**

**Response:** A box for eligible participants has been added to the CONSORT Flow chart before the ‘randomized” box.

**(9c)** **Given the detailed summary about the two-phase recruitment, I recommend separating the top two boxes by phases. Eligible participants in both phases are combined into the Randomized box.**

**Response:** The top two boxes of the CONSORT Flow chart have been separated by recruitment phase see figure below (section highlighted in yellow).

The revised CONSORT Flow chart has been submitted as Figure 1, and an image is also provided below.

**Reviewer #4:**

**1. Line 87 and 104: referencing should be kept uniform across paper**

**Response:** All in-text citations reading author and colleagues have been revised to author et al. throughout the manuscript.

**2. 75 and 83: is it BIs or BI. keep it uniform its used differently in introduction section**

**Response:** The authors have revised the terms to ensure uniformity and clarity. Brief interventions (BIs) are now used to broadly refer to the group of interventions whose goal is to motivate people to reduce harmful use, and whose content and duration can vary. A definition of BIs has been provided in the introduction section on page 3 line 76-80.

“Brief interventions (BIs) for substance use are recommended as a public health strategy for prevention and early intervention for addressing youth substance use problems (14,15). BIs typically comprise of screening to determine level of substance involvement, followed by counseling based on motivational interviewing principles (16). The intervention typically lasts 1-4 sessions (14,17).”

The term single-session brief intervention (SSBI) now refers to the specific BI which was evaluated for in this study. A brief description of the trial brief intervention which is a single-session brief intervention is provided in the introduction (page 5 line 127-130)

“The single-session brief intervention was adapted from the WHO Alcohol, Smoking & Substance Use Involvement Screening Test (ASSIST)-linked BI manual for use in primary care and is based on Feedback Responsibility Advice Menu of options Empathy Self-efficacy (FRAMES) model and motivational interviewing principles.”

A detailed description of the single session brief intervention if provided in the methods section page 11 lines 266-288 and Table 1 page 13.

**3. Table 1: as paper is written in past tense, table should also follow that. Presently its in future tense**

**Response:** Table 1 describes predefined feasibility benchmarks and does not report results, and the authors believe it is described in the appropriate tense.

**4. Line 203: Sample size calculation needs to be explained more. from where n=50 came?
please mention inclusion and exclusion criteria under a single heading, at present its in different places.**

**Response:** The sample size is informed by published sample size recommendations for pilot RCTs. According to Whitehead et al, for a main trial designed with 90% power and two-sided 5% significance, a small, standardized effect size (0.2), the recommended pilot trial sample sizes per treatment arm is **25.** The citation is below and has also been provided in the manuscript.

Citation: Whitehead AL, Julious SA, Cooper CL, Campbell MJ. Estimating the sample size for a pilot randomized trial to minimise the overall trial sample size for the external pilot and main trial for a continuous outcome variable. Stat Methods Med Res. 2016 Jun;25(3):1057-73. doi: 10.1177/0962280215588241. Epub 2015 Jun 19. PMID: 26092476; PMCID: PMC4876429.

We have included a paragraph that describes the rationale for the sample size on page 7 line 172-177.

“We aimed to have 25 participants per group (Total N=50). This sample size is large enough to inform us about the practicalities of delivering the control and the SSBI, and is in line with existing pilot RCT recommendations for proportionality to planned definitive RCT(41) (i.e., we have ≥90% power to detect a small, standardized effect size of 0.2 [an effective SSBI will result in a 20% difference in the reduction in the total ASSIST-Y scores between SSBI and psychoeducation arms]) (Whitehead et al,)”

**5. Why was 2 phase recruitment approach taken?**

**Response:** We conducted a second phase of recruitment because we did not meet minimum sample size during phase 1. The authors have described the two-phase recruitment and the rationale on pages 8-10 under the section labelled “**Adaptations to youth recruitment processes”**

**6. From where n=72 came in?**

**Response:** Sample size of 72 is based on attrition rates between recruitment and baseline data collection, and between baseline data collection and 3-month follow-up. This has been described in the methods section under sample size. Page 8 line 180-182.

“Because we anticipated 20% attrition between recruitment and baseline data collection, and a further 20% attrition between baseline data collection and the 3-month data collection, we targeted to recruit 36 participants per arm (total 72 participants). The attrition rates were based on a prior study testing a substance use intervention in our setting (43).”

**7. Table 1, why 3 month FU is blank for phase 1 and phase 2?**

**Response:** Table 1 describes the predefined feasibility benchmarks for the study that were defined apriori. At that point, the authors did not know that a two-phase recruitment would happen, so indicating phase 1 and 2 may not be applicable for this table.

**8. line 329...is using term clients suitable here?**

**Response:** The authors have replaced the term ‘clients’ with ‘youth’ lines 276-284

**9. Line 649-652: how do we define these small/ moderate/ medium positive effects?? what is the definition?**

**Response:**

The absolute value of the SMD indicates the strength of the effect, with larger values reflecting stronger effects. An SMD of (0.2) indicates a small effect, (0.5) a moderate effect, and (0.8) or higher a large effect.

We have provided these definitions in the methods section Page 19 lines 429-431.

“For interpretation in our analysis, we followed Cohen's guidelines: The absolute value of the SMD indicates the strength of the effect, with larger values reflecting stronger effects. Therefore, an SMD of (0.2) indicates a small effect, (0.5) a moderate effect, and (0.8) or higher a large effect (50). In this context, SMDs near zero suggest balanced characteristics across groups, while larger values reflect practical differences, enhancing understanding of the SSBI's potential impact and guiding future studies (51)”.

**Reference below:**

Cohen J. Statistical Power Analysis for the Behavioral Sciences (2nd ed.). Routledge. https://doi.org/10.4324/9780203771587. 1988.

**10. Overall: About analysis**- can be presented in a better way. Looks little unclear at certain points.

**Response:** The revisions above have added clarity to the analysis, which should address this concern.
